# Correction: SSNdesign—An R package for pseudo-Bayesian optimal and adaptive sampling designs on stream networks

**DOI:** 10.1371/journal.pone.0341916

**Published:** 2026-01-27

**Authors:** Alan R. Pearse, James M. McGree, Nicholas A. Som, Catherine Leigh, Paul Maxwell, Jay M. Ver Hoef, Erin E. Peterson

In Table 1, there are errors in the formulas for some of the utility functions such as CP-optimality, CPD-optimality, K-optimality, EK-optimality, Sequential CP-optimality, Sequential D-optimality, Sequential ED-optimality, the Maximin utility, and the Morris-Mitchell utility. There is also an error in Equation (17) of the S1 Appendix for the Morris-Mitchell utility. Please see the correct Table 1 here. The changes are explained by a document, S1 File, attached to this correction notice. The document also presents annotated code from the SSNdesign R package to show that no results are affected and that the correct versions of the utility functions were implemented in the first instance.

**Table 1:** Utility functions implemented in SSNdesign.

Name	Purpose	Application	Empirical	Definition of the utility	Reference
CP-optimality	Covariance parameters	OP	×	$\begin{array}{c} \text{log}\;\text{det}\left[I(\theta \right)] \end{array}$	[16]
D-optimality	Fixed effects parameters	OP	×	$\begin{array}{c} \text{log}\;\text{det}\left[\text{Var}{\left({\hat{\beta }}_{\text{gls}}\right)}^{-\text{1}}\right] \end{array}$	[15, 16]
ED-optimality	Fixed effects parameters	OP	✓	$\begin{array}{c} \text{log}\;\text{det}\left[\hat{\text{Var}}{\left({\hat{\beta }}_{\text{gls}}\right)}^{-\text{1}}\right] \end{array}$	[15, 16]
CPD-optimality	Fixed effects and covariance parameters, a mixture of CP- and D-optimality	OP	×	$\begin{array}{c} \text{log}\;\text{det}\left[\text{Var}{\left({\hat{\beta }}_{\text{gls}}\right)}^{-\text{1}}\right]+\text{log}\;\text{det}\left[I(\theta \right)] \end{array}$	
K-optimality	Predictions	OP, AD	×	${\left(\sum_{s_{z}\in S}\sigma_{\text{UK}}^{\text{2}}(s_{z})\right)}^{-\text{1}}$	[15, 16]
EK-optimality	Predictions	OP, AD	✓	${\left(\sum_{s_{z}\in S}{\hat{\sigma }}_{\text{UK}}^{\text{2}}(s_{z})\right)}^{-\text{1}}$	[15, 16]
Sequential CP-optimality	Covariance parameters	AD	×	$\begin{array}{c} \text{log}\;\text{det}\left[I(\theta )+O_{t}(\theta )\right] \end{array}$	S1 Appendix
Sequential D-optimality.	Fixed effects parameters	AD	×	$\begin{array}{c} \text{log}\;\text{det}\left[\text{Var}{\left({\hat{\beta }}_{\text{gls}}\right)}^{-\text{1}}+O_{t}(\theta )\right] \end{array}$	S1 Appendix
Sequential ED-optimality.	Fixed effects parameters	AD	✓	$\begin{array}{c} \text{log}\;\text{det}\left[\hat{\text{Var}}{\left({\hat{\beta }}_{\text{gls}}\right)}^{-\text{1}}+O_{t}(\theta )\right] \end{array}$	S1 Appendix
Maximin.	Space-filling, with an emphasis on increasing the minimum distance between pairs of points	OP	n/a	$\underset{i\neq j}{\text{min}}D(s_{i},s_{j})$	[32]
Morris-Mitchell	Space-filling, with an emphasis on increasing separations larger than the minimum	OP	n/a	$-{\left(\sum_{w=\text{1}}^{W}J_{w}D_{w}^{-p}\right)}^{\text{1}/p}$	[31]

In the Application column, OP stands for ‘optimal design’, and AD stands for ‘adaptive design’. In the Empirical column, × means No; ✓ means Yes; and n/a means ‘not applicable’. Here, θ is a vector of covariance parameters from a geostatistical model; I(θ) denotes the expected Fisher Information Matrix of the covariance parameters; ${\hat{\beta }}_{\text{gls}}$ denotes the estimates of the fixed effects; $\text{Var}\left({\hat{\beta }}_{\text{gls}}\right)$ is the covariance matrix of the fixed effects; $\sigma_{\text{UK}}^{\text{2}}(s_{z})$ denotes the universal kriging (UK) variance at prediction site $s_{z}$, and S  is the set of all prediction sites; $O_{t}(\theta )$ represents a summary statistic from the existing design after t previous design steps; $D\left(s_{i},\;s_{j}\right)$ is the distance between sites $s_{i}$ and $s_{j}$ in a design, which can be measured as Euclidean distance or hydrological distance along the stream network [10]; for w=1, ..., W, $D_{w}$ is the w-th smallest of the W  unique non-zero distances between pairs of sites in a design, and $J_{w}$ is the number of unique pairs of sites separated by the distance $D_{w}$; and p≥1 is a weighting power. Both $\text{Var}\left({\hat{\beta }}_{\text{gls}}\right)$ and $\sigma_{\text{UK}}^{\text{2}}\left(s_{z}\right)$ depend on covariance parameters θ. In the empirical utility functions, the covariance parameters are estimated from data simulated using the prior draws (see [16]), and the “hats” on $\hat{\text{Var}}\left({\hat{\beta }}_{\text{gls}}\right)$ and ${\hat{\sigma }}_{\text{UK}}^{\text{2}}(s_{z})$ indicate the estimated covariance parameters were used to compute these quantities.

## Supporting information

S1 File Responses to an editor’s comments on the corrected Table 1, with annotated code. (PDF)
