# Supplementary material for: SSNdesign—An R package for pseudo-Bayesian optimal and adaptive sampling designs on stream networks
Source: PLoS One. 2020 Sep 22;15(9):e0238422. doi: 10.1371/journal.pone.0238422 (PMC7508409; doi:10.1371/journal.pone.0238422)
Supplement: S3 Appendix — (PDF) [file pone.0238422.s003.pdf]

S3 Appendix: Glossary of terms

Alan R. Pearse, James M. McGree, Nicholas A. Som, Catherine Leigh, Paul Maxwell, Jay M. Ver Hoef, Erin E. Peterson

2020-07-08

Glossary

Adaptive design A framework for building up or modifying a monitoring program over time. This framework uses data that has already been collected in an existing monitoring program to inform the next decision(s) about new sites to sample in the next sampling period.

Bayesian statistics A treatment of statistical uncertainty developed by Reverend Thomas Bayes. This approach to uncertainty is characterised by the use of prior information and new observations and data together to construct posterior distributions around parameters of interest. See also **pseudo-Bayesian**.

Correlation The relatedness of two observations or variables. Things that share a larger correlation share a stronger bond between them. Correlations are normalised between -1 (perfect inverse relationship) and 1 (perfect positive relationship), with a 0 indicating a complete lack of a relationship.

Covariance The relatedness of two observations or variables. Things that share a larger covariance share a stronger bond between them. Covariances are unnormalised **Correlations**. That is, they do not necessarily range between -1 and 1; instead, they can range between negative and positive infinity.

Covariance function A mathematical function that describes the relatedness of any two or more observations in space in terms of only the distance between the spatial locations of the two observations. Covariance functions tend to have three parameters: the **Nugget effect**, the **Range**, and the **Sill**.

Covariate Any variable that is thought to affect the outcome of an **Experiment**. These variables are also measured or recorded alongside the **Response variable** during the experiment so that the relationship between them can be quantified.

Design A particular configuration of an experiment. In the context of **Stream networks** and **Spatial statistics**, a design is a set of **Sites** that are sampled as part of a monitoring program.

Design criterion See **Utility function** and **Expected utility**.

Efficiency The amount of information gained from unit effort in an experiment. More efficient experiments need fewer samples to gain the same amount of information as less efficient experiments. See also **Relative efficiency**.

Estimate A statistically informed guess about the value of an unobserved **Parameter** in a **Model** or a population based on incomplete data.

Euclidean distance Distance as the crow flies. That is, for two points A and B in 2-dimensional space, Euclidean distance is just the length of the line that separates the two.

Expected utility Qualitatively, this is a measure of how fit a **Design** is for a user-specified purpose. Higher values of the expected utility indicate higher suitability. Mathematically, it is the average value of the **Utility function** once all the parameters and data have been accounted for.

Experiment A scientific investigation carried out under controlled or observational conditions where the investigator collects some data about a process of interest.

Experimental design The deliberate construction of experiments, usually controlled experiments but also observational experiments, in a way that makes the observed data easier to statistically analyse and may also strengthen any statistical conclusions drawn from those data.

Fisher information (matrix) For a single **Parameter**, the Fisher information is a single number that describes how much uncertainty there is in the estimated value of that parameter. For multiple parameters, the Fisher information is instead a matrix where the elements on the diagonal describe the uncertainty in the parameters and the off-diagonal elements describe how related the parameter estimates are to each other.

Frequentist statistics A framework for statistics where model parameters are estimated from observed data only and no prior beliefs about the parameters are incorporated into the estimates. Most conventional models in statistics are frequentist.

Geostatistics A field of statistics for fitting models to data collected at different locations in space, where the observed data themselves (as opposed to the locations of the data) are of primary interest.

Kriging A method of making statistically rigorous predictions of a variable of interest to unsampled locations in space.

Kriging variance The uncertainty of a *Prediction* made using *Kriging*.

Likelihood A mathematical function that describes the distribution of the data one has observed during an experiment.

Loss function A function to be minimised. Loss functions typically describe error; for example, discrepancies between a modelled value and an observed value.

Model Usually a smooth line which relates an outcome variable to values of several input variables. It has several **Parameters**, which typically quantify the effect that each input variable has on the outcome variable.

Myopic design approach In the context of **Adaptive design**, this is a framework for building an experimental design in a series of steps where one tries to optimise decisions about the placement of sites for a single step in the future.

Nugget effect The nugget effect is the natural variation observed between observations taken at exactly the same location.

Optimal design An **Experimental design** that has the highest value of the **Expected utility** among a set of other potential designs. If the definition of the expected utility is carefully constructed, then being the optimal design may have several advantages; for example, providing the least uncertain **Parameter estimates** in a **Model**.

Optimality The state of having achieved the best possible value of a function. In the context of **Experimental design**, this means either having maximised a **Utility function** or the **Expected utility**.

Parameter An unobserved value in a **Model**. These are usually unknown *a priori* and must instead be inferred from the data.

Posterior In Bayesian statistics, the posterior is a distribution that represents one's knowledge about some parameter(s) of a system as a combination of one's subjective prior belief and data that they have recently observed.

Prediction The modelled value of a variable of interest at an unsampled location.

Prior In **Bayesian statistics**, a prior is typically 'set' on a **Parameter** in a **Model** to summarise one's existing subjective beliefs or knowledge about the distribution of that parameter. A prior is usually expressed as a distribution but it can also be a single value (this is called a point prior, but it is generally avoided because this is a statement made with extreme certainty that a parameter has a

specific value). The prior is updated by the data one collects as part of one's experiment(s) to yield the **Posterior**.

Pseudo-Bayesian statistics A framework for statistics that is neither fully frequentist or Bayesian.

Random design In the context of **Stream networks** and **Spatial statistics**, this refers to a collection of randomly chosen locations on a stream that are sampled for data. These designs are a helpful benchmark because optimal and adaptive designs, which are very deliberately constructed, should at least outperform randomly chosen designs in the task they have been optimised for.

Range The range is the distance at which any pair of observations no longer share an intrinsic spatial relationship. Expressed differently, if two sites A and B are separated by a distance greater than the range, they are far enough apart that observing something at site A gives you no information about what might be going on at site B.

Relative efficiency The ratio of the information gained from two different experimental configurations (typically including the same number of observations). This figure indicates how many times more sites are needed in the less efficient design to yield the same information as the more efficient design.

Response variable The outcome or dependent variable in an experiment. Explicitly interpreted for stream health monitoring programs, examples of response variables include dissolved oxygen, macroinvertebrate diversity and/or indices, and in-stream temperature.

Sequential design See **Adaptive design**. This terminology is avoided as much as possible in this work, but is common in the broader experimental design literature.

Sill The sill is the variance that two unrelated observations will have.

Sites A location on a stream network where some measurement about processes occurring in the stream are taken. The measurement(s) may relate, for example, to water quality, to biodiversity, or to flow height and/or volume.

Spatial autocorrelation The relatedness of observations that are separated by distance in space.

Spatial statistics An area of study within statistics that is concerned with characterising and exploiting the relatedness of data collected in space. Relatedness typically increases the closer two points in space are to each other, and decreases with growing distance between them.

Stream network A stream network is a system of waterways that flow into each other. For the purposes of defining statistical models on stream networks, only dendritic (branching) networks where there are no braids and no more than two streams flow into a single juncture are considered.

Stream network distance Distance as measured by tracing the length of a **Stream network** that separates two points. The network distance between two points must be equal to or greater than the **Euclidean distance** between two points in space.

Space-filling design A configuration of sampling sites that ensures the maximum and most uniform spatial coverage from a fixed number of sites.

Static design See **Optimal design**.

True model A *Model* that is believed to adequately describe a process that one is sampling and observing during an *Experiment*. Note, however, that, philosophically, the notion of a 'true model' is tenuous; it is very rare that the underlying process is fully described by a mathematical function.

Utility function A function to be maximised. Contrast to **Loss function**.

Variable Any process or thing that can be measured. Some variables for stream surveys may include dissolved oxygen, water temperature, air temperature, canopy cover, width of the stream at the sampling site.

Variance The uncertainty inherent in a variable or observation. Higher variances indicate higher uncertainties.
