# Supplementary material for: SSNdesign—An R package for pseudo-Bayesian optimal and adaptive sampling designs on stream networks
Source: PLoS One. 2020 Sep 22;15(9):e0238422. doi: 10.1371/journal.pone.0238422 (PMC7508409; doi:10.1371/journal.pone.0238422)
Supplement: S2 Appendix — (PDF) [file pone.0238422.s002.pdf]

S2 Appendix: SSNdesign - an R package for pseudo-Bayesian optimal and adaptive sampling designs on stream networks

Alan R. Pearse, James M. McGree, Nicholas A. Som, Catherine Leigh, Paul Maxwell, Jay M. Ver Hoef, and Erin E. Peterson

2020-07-08

The SSNdesign package

The `SSNdesign` package provides functions to find pseudo-Bayesian optimal and adaptive designs for spatial stream network models and streams data. Given a set of potential sampling locations on a stream network and a utility function (i.e. mathematical statement about the sampling objective), SSNdesign will find the best subset of sites to meet that objective. This vignette steps through two case studies of stream sampling problems that can be solved using `SSNdesign`. These involve:

1. Using optimal design to reduce the number of sites in a monitoring program by half, using data collected near Lake Eacham, Queensland; and
2. Augmenting an existing monitoring program with new sites within the Pine River catchment, Queensland, using adaptive design.

The data required for this tutorial are downloaded with the package. Instructions for extracting these files are provided below.

Installing and loading the package

The package can be installed by running `devtools::install_github("apear9/SSNdesign")`. If the package files have already been downloaded from GitHub and are stored in a local directory, the package can also be installed by running `devtools::install_local(path)` where `path` is a string specifying the location of the directory containing the package files. For Windows users, `devtools::install_local` requires Rtools. Once installed, the package can be loaded by running

```r
library(SSNdesign)
```

Extracting the example data from the package

Once installed and loaded, the example data required for this tutorial can be extracted from the package by running the following code.

```r
# The ... should be replaced with the path to an empty,
# new folder you have set up to receive the package data.
setwd("...")
unpackExampleData(".") # this should return TRUE
```

Case study 1: Lake Eacham

The Lake Eacham dataset contains stream temperature measurements collected at 88 sites throughout a stream network in northern Queensland, Australia. Each site is also associated with a set of GIS-derived covariates such as total rainfall (mm) on the day of sampling, percent urban, grazing and agricultural land-use within the watershed, stream slope, and the distance upstream of the outlet (i.e. the most downstream location on the stream network). In this case study, the goal is to reduce the number of sampling sites from 88 to 44, while retaining the sites that yield the most information. This is a classic use-case for optimal experimental design because there is one decision to be made at a single point in time (i.e. which 44 sites should be dropped from the monitoring program after this year). We can optimise this design for a number of objectives, but two common objectives that concern managers are to (1) characterise in-stream processes using a statistical model and (2) accurately predict in-stream variables throughout the stream network at unobserved locations. For the first objective, we optimise the design using CPD-optimality, which is a utility function that aims to minimise uncertainty in the fixed effect and covariance parameters produced by the geostatistical model. For the second objective, we optimise the design using K-optimality. This utility function aims to maximise prediction accuracy at unobserved locations by reducing the average uncertainty across prediction sites in the stream network. In this section, we demonstrate how to solve this problem using `SSNdesign`.

The first step in any design procedure using `SSNdesign` is to import a `SpatialStreamNetwork` object. This object already contains streams, observed sites with measurements. Thus, we simply use `importSSN` from the package `SSN` to load a SpatialStreamNetwork object into R. A dataset of prediction sites is also imported, which will be needed to construct a design that focuses on maintaining prediction accuracy at unsampled sites.

```r
# import spatial stream network
lake.eacham <- importSSN("lake-eacham-full.ssn", predpts="preds")

# create distance matrices for obs and preds sites
createDistMat(lake.eacham, predpts="preds", o.write=TRUE, amongpreds=TRUE)
```

We can check what this stream network looks like using the plot method for `SpatialStreamNetwork` objects.

```r
# plot the network
plot(lake.eacham, "Temp")
```

A prerequisite of optimal design is that we are able to define a 'true' spatial statistical model. The notion of a 'true' model refers to a model that we believe adequately describes the process responsible for generating the data. Designs are then optimised in relation to some element of this true model (e.g. the parameters and/or predictions). Note that we only need to know the structure of the model, which, for spatial stream-network models, includes the mean structure for the fixed effects and the covariance function for the covariance parameters. In our case, we assume that the mean structure for temperature is a function of total rainfall (mm) and the percent of urban and grazing land use in the riparian zone. We also include the exponential tail up and tail down components in the covariance mixture. We then fit this model to our data. Note that fitting the model does not necessarily mean the estimates from the model are used when optimising designs using `optimiseSSNDesign`. The main reason for fitting this model is to give `optimiseSSNDesign` a template for its own operations, such as constructing design and covariance matrices when evaluating a utility function.

```r
# Fit the 'true' model to the data
TC.model <- glmssn(
  Temp ~ rainfall + ripURBAN + ripGRAZE,
  lake.eacham,
```

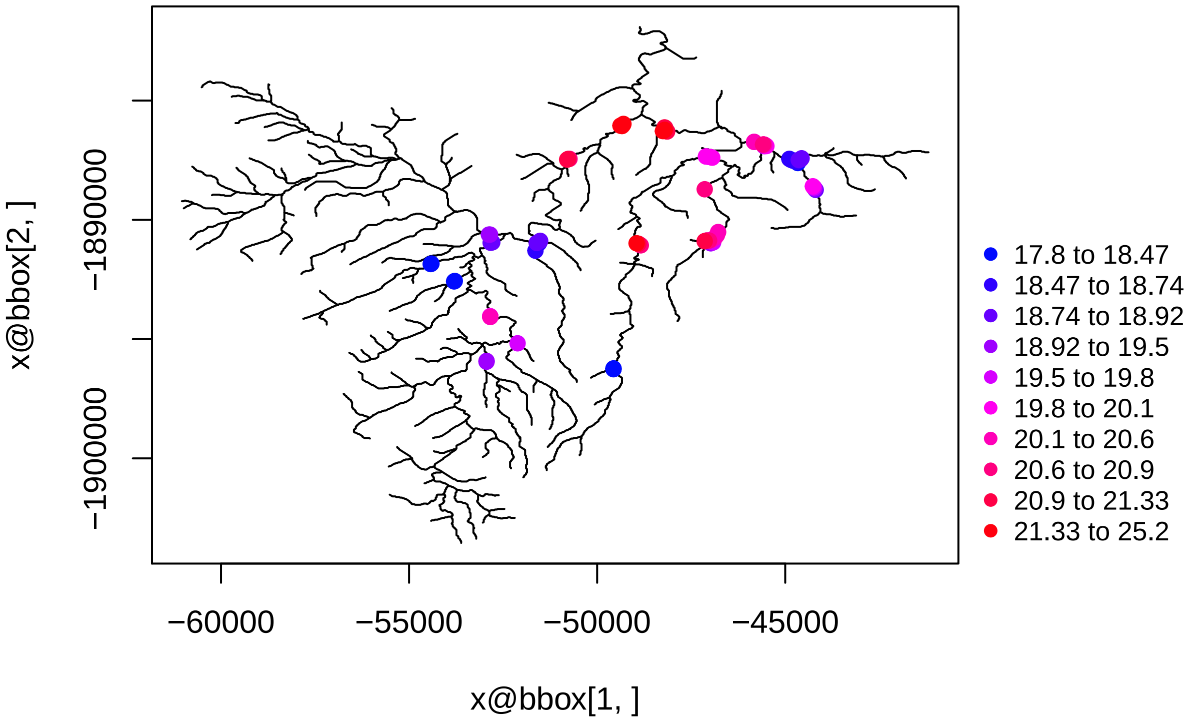

Figure 1: The Lake Eacham stream network with sampling points.

```r
  CorModels = c("Exponential.tailup", "Exponential.taildown"),
  addfunccol = "afvArea"
)
```

We define log-normal priors on the covariance parameters based on the fitted model.

```r
# Create a list of functions used to define log-normal priors based on the
# estimates of the covariance parameters and their standard errors.
priors.TC <- constructLogNormalPriors(TC.model)
```

The next step is to optimise the design. We show the code for the CPD-optimal design first. Note that, the `optimiseSSNDesign` function could be used to find the optimal 44-site designs from a few (5 - 10) random starts. However, in this case study the aim is to remove sites from an existing monitoring program one-by-one, so that we can quantify how much information is lost as sites are removed.

Finding an optimal design using the functions `CPDOptimality` or `KOptimality` is often computationally expensive. We have provided the R code below so that users can recreate the examples using their own data. However, we also provide the results as saved Rdata workspaces, which can be found in the Supplementary Information and loaded into R to save time.

```r
# Find the CPD-optimal design for 44 of the 88 sites
# WARNING: this code takes approximately 5 hours to run.

# # Set random seed for reproducibility
# set.seed(987654321)

# # Initialise loop by dropping the first site
# step88to87 <- optimiseSSNDesign(
#   ssn = lake.eacham,
#   new.ssn.path = "./to87CPD.ssn",
#   glmssn = TC.model,
#   n.points = 87,
#   utility.function = CPDOptimality,
#   prior.parameters = priors.TC,
#   n.cores = 1,
#   parallelism = "none",
#   n.optim = 1,
#   n.draws = 500
# )
# createDistMat(step88to87$ssn.new)

# # Loop through the remaining steps dropping sites one-by-one
# indices <- 86:44
# counter <- 1
# n.indices <- length(indices)
# cpd.designs <- vector("list", n.indices + 1)
# cpd.designs[[1]] <- step88to77$final.points

# for(i in indices){
#   current.pth <- paste0("./to", i+1,"CPD.ssn")
#   ifuture.pth <- paste0("./to",i,"CPD.ssn")
#   current.ssn <- importSSN(current.pth)
#   current.ssd <- optimiseSSNDesign(
```

```
#     ssn = current.ssn,
#     new.ssn.path = ifuture.pth,
#     glmssn = TC.model,
#     n.points = i,
#     utility.function = CPDOptimality,
#     prior.parameters = priors.TC,
#     n.cores = 1,
#     parallelism = "none",
#     n.optim = 1,
#     n.draws = 500
#   )

#   createDistMat(current.ssd$ssn.new)
#   cpd.designs[[counter + 1]] <- current.ssd$final.points
#   counter <- counter + 1
# }
# save.image("CPD-OPTIMAL-RESULTS.Rdata")

# Load the results from the CPD-optimal design process
load("CPD-OPTIMAL-RESULTS.Rdata")
```

The process for finding the K-optimal design is very similar. The differences are that

- We need to use the `KOptimality` utility function instead of `CPDOptimality`.
- The `SpatialStreamNetwork` object must contain prediction sites.
- Distance matrices must be generated for both the observed and prediction sites when the `SpatialStreamNetwork` object is imported.

```
# Find a K-optimal design for 44 of the 88 sites
# Warning, this code may take up to 5 hours to run

# # Set random seed for reproducibility
# set.seed(123456789)
#
# step88to87 <- optimiseSSNDesign(
#   ssn = lake.eacham,
#   new.ssn.path = "./to87K.ssn",
#   glmssn = TC.model,
#   n.points = 87,
#   utility.function = KOptimality,
#   prior.parameters = priors.TC,
#   n.cores = 1,
#   parallelism = "none",
#   n.optim = 1,
#   n.draws = 500
# )
# createDistMat(step88to87$ssn.new, "preds", T, T)
#
# # Loop to find optimal designs using one-dimensional optimisation
# indices <- 86:44
# counter <- 1
```

```r
# n.indices <- length(indices)
# k.designs <- vector("list", n.indices + 1)
# k.designs[[1]] <- step88to77$final.points

# for(i in indices){
#   current.pth <- paste0("./to", i+1,"K.ssn")
#   ifuture.pth <- paste0("./to",i,"K.ssn")
#   current.ssn <- importSSN(current.pth, "preds")
#   createDistMat(current.ssn, "preds", T, T)
#   current.ssd <- optimiseSSNDesign(
#     ssn = current.ssn,
#     new.ssn.path = ifuture.pth,
#     glmssn = TC.model,
#     n.points = i,
#     utility.function = KOptimality,
#     prior.parameters = priors.TC,
#     n.cores = 1,
#     parallelism = "none",
#     n.optim = 1,
#     n.draws = 500
#   )
#   createDistMat(current.ssd$ssn.new, "preds", TRUE, TRUE)
#   k.designs[[counter + 1]] <- current.ssd$final.points
#   counter <- counter + 1
# }
# save.image("K-OPTIMAL-RESULTS.Rdata")

# Load the results from the K-optimal design process
load("K-OPTIMAL-RESULTS.Rdata")
```

The output of `optimiseSSNDesign` is an S3 object of class `ssndesign`. This is a list of 14 elements that contains the `SpatialStreamNetwork` object passed to it, a modified version containing only the observed sites associated with the optimal or adaptive design, and diagnostic information associated with the Greedy Exchange Algorithm it uses to find optimal and adaptive designs. Additionally, objects of class `ssndesign` contain information about the user's call to `optimiseSSNDesign`, including, for example, the values of key parameters and also the prior draws that were used in the Monte Carlo integration.

Having computed an optimal design, the next step is to check that it performs well compared to random and Generalised Random Tessellation Sampling (GRTS) designs. We chose to benchmark our solution against GRTS designs because they are a powerful tool for constructing spatially balanced designs. However, any standard design (e.g. random sampling) can be chosen as a benchmark.

In practical terms, validating a design using the functions in `SSNdesign` requires the following:

1. Finding one or more optimal design(s) and recording the sites included in each design.
2. Identify designs of the same size as the optimal design to benchmark against. Here, size refers to either the number of sites or number of samples collected per sampling period. If it makes sense to compare the optimal design against other designs of different sizes then this is also possible.
3. Evaluating the expected utility of the benchmarking designs with a large number of Monte-Carlo draws and then computing the designs' relative efficiency compared to the optimal design.

The first step was completed in part when we found a series of optimal designs. We must now record the sites included in those designs, which we can do with the following code:

```r
# Record designs for CPD-optimality

# Set up an empty vector to store the designs
# Designs are stored as a list of vectors containing pid or locID values
opt.cpd.reference <- append(
  list(1:88), # the full desig with all 88 sites
  cpd.designs
)

# Record designs for K-optimality, repeating the same process
opt.k.reference <- append(
  list(1:88), # the full design
  k.designs
)

# Save the output as a .Rdata file
# save(opt.cpd.reference, opt.k.reference, file = "optimal-designs.Rdata")
```

The second step is to set up the GRTS and random benchmarking designs.

```r
# The following code takes a few minutes to run.
# If you do not run the following code, you must run
# load("reference-designs.Rdata")
# before continuing to the next code block

# Need to reset the path for lake eacham data
lake.eacham <- updatePath(
  lake.eacham,
  # We need the full path
  paste(getwd(), "lake-eacham-full.ssn", sep = "/")
)

# Seed for reproducibility
set.seed(1)

# Set up an empty vector to store the GRTS designs
grts.reference <- vector("list", 20)
# Note: the following loop takes approximately
# 10-15 minutes to run.
for(i in 1:20){
  # For each iteration, we
  # 1. Create a GRTS design using drawStreamNetworkSamples
  # 2. Record the pid values associated with the GRTS design
  g <- drawStreamNetworkSamples(
    lake.eacham, paste0(tempdir(), "/g", i, ".ssn"), T, "GRTS", 44
  )
  grts.reference[[i]] <- getSSNdata.frame(g)$pid
}

# Set up an empty vector to store random designs
rand.reference <- vector("list", 20)
for(i in 1:20){
  # Simply store a random sample (w/out replacement)
```

```r
  # of 44 out of 88 possible pid values
  rand.reference[[i]] <- sample(1:88, 44, FALSE)
}

# Save info in .Rdata file
# save(rand.reference, grts.reference, file = "reference-designs.Rdata")
```

The final step is to evaluate the expected utility for each of these designs using a large number of Monte Carlo draws.

```r
# The following code may take an hour to run.
# If you do not run the following code, you must run
# load("optimal-info.Rdata")
# load("rand-grts-info.Rdata")
# before continuing to the next code block

# Reset path to lake.eacham in case overwritten by loaded .Rdata file
lake.eacham <- updatePath(
  lake.eacham,
  # We need the full path
  paste(getwd(), "lake-eacham-full.ssn", sep = "/")
)

# Set seed for reproducibility
set.seed(1e6 + 1)

# Evaluate the expected utility of the designs discovered
CPD_info <- evaluateFixedDesigns(
  lake.eacham, TC.model, opt.cpd.reference,
  "pid", CPDOptimality, priors.TC, 1000
)
K_info <- evaluateFixedDesigns(
  lake.eacham, TC.model, opt.k.reference,
  "pid", KOptimality, priors.TC, 1000
)
# save(CPD_info, K_info, file = "optimal-info.Rdata")

# Evaluate the 44-site random and GRTS design
R_CPD_info <- evaluateFixedDesigns(
  lake.eacham, TC.model, append(grts.reference, rand.reference),
  "pid", CPDOptimality, priors.TC, 1000
)
R_K_info <- evaluateFixedDesigns(
  lake.eacham, TC.model, append(grts.reference, rand.reference),
  "pid", KOptimality, priors.TC, 1000
)
# save(R_CPD_info, R_K_info, file = "rand-grts-info.Rdata")
```

Note that `evaluateFixedDesigns` has a `data.frame` output, which looks like this:

```r
# for the reference designs under CPD optimality
head(R_CPD_info)
#>   ID Size Expected utility Efficiency Efficiency_Unlogged
```

```
#> 1  1   44         -21.31065   1.052198          0.3474338
#> 2  2   44         -20.61476   1.017839          0.6967726
#> 3  3   44         -20.69756   1.021927          0.6414036
#> 4  4   44         -20.25347   1.000000          1.0000000
#> 5  5   44         -21.32713   1.053011          0.3417535
#> 6  6   44         -20.86973   1.030428          0.5399558
```

Notice here that there are columns giving the expected utility of each design, and also the efficiency of each design *relative to the design with the highest expected utility in the* ***data.frame***. The problem with this is that we have computed the expected utilities of the optimal and reference designs separately. Therefore, an additional step is needed at this juncture to compute the efficiencies of the reference designs compared to the full 88-site design.

```
# Evaluate relative efficiencies
R_CPD_info$Type <- rep(c("GRTS", "Random"), each = 20)
R_CPD_info$E2 <- exp(
  R_CPD_info$`Expected utility` - max(CPD_info$`Expected utility`)
) # Compute efficiency relative to full design
R_K_info$Type <- rep(c("GRTS", "Random"), each = 20)
# Compute efficiency relative to full design
R_K_info$E2 <- R_K_info$`Expected utility`/max(K_info$`Expected utility`)
```

Now that we have computed the relative efficiencies, we can plot the summaries. We have chosen to use ggplot2.

```
library(ggplot2)
library(gridExtra)

a <- ggplot(data = CPD_info, aes(x = Size, y = Efficiency_Unlogged)) +
  geom_path() +
  geom_jitter(data = R_CPD_info,
              aes(x = 44, y = E2, col = Type), size = 4, alpha=0.4) +
  ylim(c(0, 1)) +
  labs(x = "Number of sampling sites", y = "Efficiency", title = "(a)") +
  scale_x_reverse() +
  theme_bw() +
  theme(legend.position = c(0.2, 0.2),
        legend.background = element_rect(colour = "black"),
        text = element_text(size = 16))

b <- ggplot(data = K_info, aes(x = Size, y = Efficiency)) +
  geom_path() +
  geom_jitter(data = R_K_info,
              aes(x = 44, y = E2, col = Type), size = 4, alpha=0.4) +
  ylim(c(0, 1)) +
  labs(x = "Number of sampling sites", y = "Efficiency", title = "(d)") +
  scale_x_reverse() +
  theme_bw() +
  theme(legend.position = c(0.2, 0.2),
        legend.background = element_rect(colour = "black"),
        text = element_text(size = 16))

c <- ggplot(data = CPD_info, aes(x = Size, y = Efficiency_Unlogged)) +
```

```
  geom_path() +
  geom_jitter(data = R_CPD_info,
              aes(x = 44, y = E2, col = Type), size = 4, alpha=0.4) +
  ylim(c(0, 0.3)) +
  labs(x = "Number of sampling sites", y = "Efficiency", title = "(c)") +
  scale_x_reverse() +
  theme_bw() +
  theme(legend.position = c(0.2, 0.2),
        legend.background = element_rect(colour = "black"),
        text = element_text(size = 16))

d <- ggplot(data = K_info, aes(x = Size, y = Efficiency)) +
  geom_path() +
  geom_jitter(data = R_K_info,
              aes(x = 44, y = E2, col = Type), size = 4, alpha=0.4) +
  ylim(c(0.7, 1)) +
  labs(x = "Number of sampling sites", y = "Efficiency", title = "(d)") +
  scale_x_reverse() +
  theme_bw() +
  theme(legend.position = c(0.2, 0.2),
        legend.background = element_rect(colour = "black"),
        text = element_text(size = 16))

grid.arrange(ncol = 2, nrow = 2, a, b, c, d)
```

This gives us the graph below. This graph shows the efficiency of our optimal designs relative to the full design (i.e. the current monitoring program with 88 sites). The black line tracks how much information we get from sampling n sites in the optimal designs for $n \in \{87, 86, ..., 44\}$ compared to sampling all the sites. The blue and red dots on the right-hand side of each plot panel represent the amount of information we get from sampling 44 randomly chosen or GRTS sites compared to the full 88-site monitoring program. The CPD- and K-optimal designs consistently outperform the random and GRTS designs. This shows the optimal design is an effective way of choosing which 44 sites to keep and which to discard, compared to two common sampling strategies.

Case study 2: Pine River

Pine River is located in South East Queensland, Australia. Its catchment is one of those monitored by the Ecosystem Health Monitoring Program (EHMP) administered by Healthy Land and Water. In this synthetic example, we demonstrate how to extend an existing monitoring program using adaptive design using the Pine River catchment as an example location. Currently, an extensive monitoring program does not exist within Pine River alone. However, we assume for the sake of illustration that a monitoring program has operated there for two years, using 200 sites, with the primary goal of measuring dissolved oxygen (DO) levels (mg/L) and predicting them throughout the stream network. Our goal is to extend this monitoring program optimally over two years by adding another 100 sites to the program. We plan to add 50 sites per year for the third and fourth year of the program. We will be using adaptive design because there is an existing design which we expect will change year-to-year depending on any new data we collect. The goal of the monitoring program is to be able to accurately predict DO levels, so it is appropriate to adaptively update our design using the K-optimality utility function.

As before, we need to import our stream network into R. In this case, we have a set of edges marking the locations of the streams but we do not have any observed sites. In this situation, we must use `importStreams` to import our .ssn folder into R and subsequently use `generateSites` and `SimulateOnSSN` to create a set

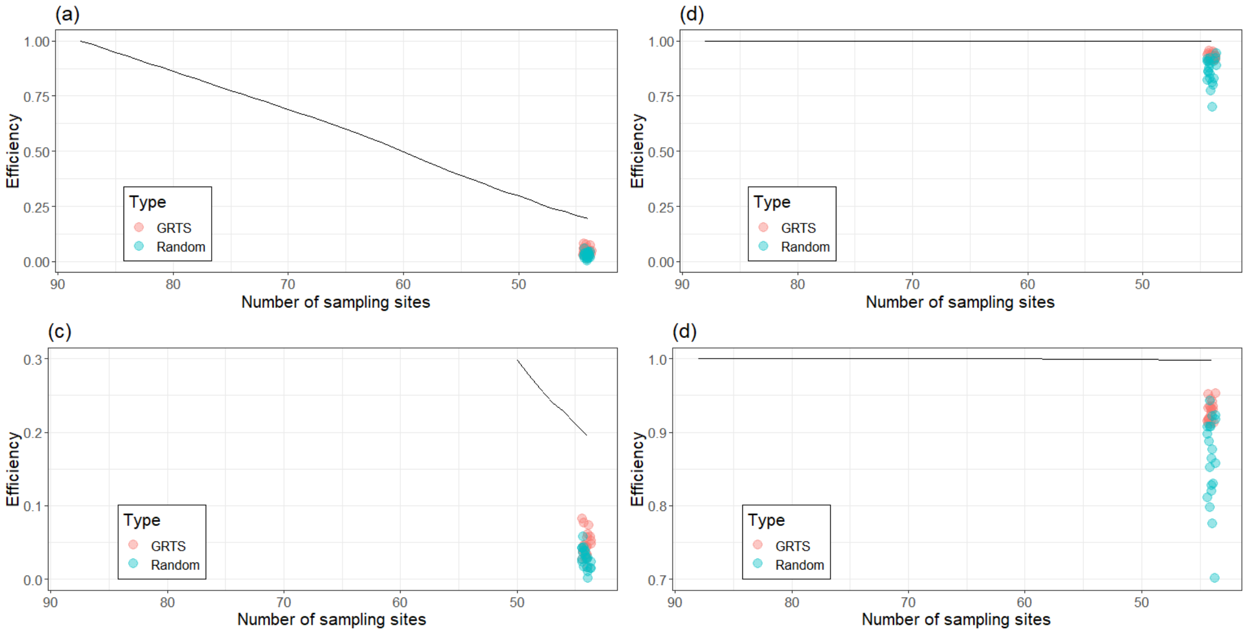

of potential sampling sites and simulate observed data on them. The code below is for the step where we generate the locations of the potential sampling sites. We have the code commented out because it takes a few minutes to run. We have provided the .ssn folder which is the output of this code block in the data.

```
# NOTES:

# before running the following code blocks, you need to run
# setwd(...) where ... is the path to the folder containing the
# data for this tutorial

# in addition, the output of several code blocks is a .ssn folder.
# we have provided these same .ssn folders with the example data.
# the code blocks may result in errors if you attempt to run them
# while the existing .ssn folders of the same name are still saved
# in your data directory. a suggested strategy is to create a new
# folder on your computer and to move the example data to that folder,
# so the resulting .ssn folders can easily be written to your working
# directory.

# import the stream network with edges only as a
# SpatialStreamNetwork object
# pine_river <- importStreams(
#   "pine_river.ssn"
# )

# put sites on this network
# with_sites <- generateSites(
#   ssn = pine_river,
#   obsDesign = systematicDesign(
#     1500,
#     replications = 4,
#     rep.variable = "Year",
```

```r
#     rep.values = 0:3
#   ),
#   predDesign = systematicDesign(1500),
#   o.write = TRUE
# )
# createDistMat(with_sites, "preds", TRUE, TRUE)
```

Now we simulate data on all 900 potential sampling sites for 4 years. That is, we are simulating the data that we would observe at any site at any point in time if we choose to sample it. We hope that this will allow us to emulate a real data collection example. The process should look like this:

1. We simulate all the data that we could possibly observe at any given site for any given year.
2. We find a 200-site GRTS design and we form our initial dataset using the simulated samples observed from these sites in the first and second years. We did not need to know anything about the stream network to find the GRTS design so this represents a scenario where we are collecting an initial sample to form our first ideas about how in-stream processes in our study area operate.
3. We use the information from the two years of sampling at our 200 GRTS sites to choose our next 50 sites adaptively.
4. We add the simulated data at our GRTS sites and our new 50 adaptive sites to our sample.
5. We update our knowledge about in-stream processes based on these new data.
6. We use all of our current information to choose the next 50 sites adaptively.

Note that, in a real situation, steps 1, 2, 4, and 6 would be slightly different. We would not simulate any data at the first step. At the second step, we would lay out the same GRTS design but instead of forming a sample by using the simulated values at those sites, we would go into the field and directly sample the GRTS sites. At the fourth step, we would again sample the GRTS sites plus the 50 sites chosen adaptively. At the sixth step, we would again sample all previously chosen sites in addition to the extra 50 sites chosen adaptively. The point here is that we only simulate data in lieu of being able to perform real data collection. Therefore, it may not be necessary to replicate this exact process of simulation for a user's own use-case.

The first step in the data simulation process is to import the .ssn folder we created before, and to extract some covariates (in particular, stream order and the additive function values) to the potential sampling sites from the stream edges.

```r
# import stream network
pine.river <- importSSN("./pine_river.ssn", "preds")

# Extract some covariates from the edges
# Start by computing Shreve Stream Orders for the edges
pine.river <- calculateShreveStreamOrderAndAFVs(pine.river)
# We need this later
pine.river@predpoints@SSNPoints[[1]]@point.data$Year <- 1
# Now extract the shreve stream order and edge AFV values to the points
pine.river <- extractStreamEdgeCovariates(
  # The SpatialStreamNetwork object
  pine.river,
  # The columns to extract from the edges data
  c("AreaAFV", "shreve")
)
```

We then compute a new covariate, which is the standardised stream order (stream order divided by maximum stream order).

```
# The following code takes approximately 2 minutes to run.
# The most computationally intensive part is creating the
# distance matrices.
pine.river <- transformSSNVars(
  # First, give the SpatialStreamNetwork object
  pine.river,
  # Then give the name of a new output folder
  # Note that in the example data the output of this function call
  # is called pine_river_sim_.ssn; the name has been changed here to
  # avoid a name conflict when writing out the result
  "pine_river_sim.ssn",
  # This is the format for creating new columns in the
  # point.data slot of the obspoints@SSNPoints[[1]] of the
  # SpatialStreamNetwork. The left-hand-side is the new column name
  # and the right-hand side is the existing column name.
  c("order" = "shreve"),
  # The same as above but not for the prediction points
  c("order" = "shreve"),
  # The function which is applied to the RHS column to create
  # the new LHS column
  function(x) x/max(x), TRUE
)
# We need to redo the distance matrix computations
# because we have created a new folder for this SSN
createDistMat(pine.river, "preds", TRUE, TRUE)

# If you didn't run the above code block, you may want to
# run
# pine.river <- importSSN("pine_river_sim_.ssn", "preds")
# createDistMat(pine.river, "preds", TRUE, TRUE)
```

This is because we expect DO to decrease with increasing stream order. Therefore, we model DO as a function of normalised stream order. We set the regression parameter for normalised stream order to -5 mg/L per unit DO. The covariance mixture included a Spherical tailup function and a random effect for each site, to account for the temporal replication which occurs as the adaptive design progresses. The partial sill, range, random effect variance and nugget parameters were assumed to be 4, 20000, 1, and 1, respectively. This finally leads us to the point of simulating the data from such a model:

```
# Set a random seed
set.seed(123)

# Simulate data on SSN
# This takes ~ 4 - 5 minutes
pine.river <- SimulateOnSSN2(
  # This function is used in exactly the same way as
  # SimulateOnSSN but all arguments must be matched
  # explicitly by name.
  ssn.object = pine.river,
  ObsSimDF = getSSNdata.frame(pine.river),
  PredSimDF = getSSNdata.frame(pine.river, "preds"),
  PredID = "preds",
  formula = ~ order,
  # calculate stream order / max(stream order),
```

```
  # insert as variable with slight negative effect
  # without random errors, this means our nominal range of
  # Dissolved Oxygen (mg/L) is 6 - 11 mg/L.
  coefficients = c(11, -5),
  CorModels = c("Spherical.tailup", "locID"),
  CorParms = c(4, 2e4, 1, 1),
  addfunccol = "AreaAFV"
)
```

An additional and non-intuitive step is needed after simulating the data. When designing a monitoring program over several years with the intention of keeping the sites from earlier time periods of sampling as legacy sites in later periods of sampling, we need to split up the observed sites shapefile by levels of the time period variable. This can be done with the following code:

```
# Split up sites by time period (year)
first.year <- splitSSNSites(
  # We're splitting this object
  pine.river,
  # Name of new .ssn folder to create
  "Year_1_.ssn",
  # What column we are splitting by
  "Year",
  # Whether we split the predictions as well
  # We only have one year of prediction sites so
  # it does not make sense to do this.
  FALSE
)
```

We can now continue with our example. In this case study, we start with two years of data collected from an established monitoring program containing 200 sites. To emulate this in our synthetic case study, we set up a GRTS design to serve as the existing monitoring program. We choose a GRTS design because the spatially balanced samples they provide are known to be efficient for prediction. In our case study, where the existing monitoring program was set up without any previously collected data, using a GRTS design for the first phase of data collection is reasonable. This is the code needed to establish the GRTS design for the first two years of sampling:

```
# Select 200-site GRTS design
first.grts <- drawStreamNetworkSamples(
  first.year,
  "Year_1_GRTS.ssn",
  TRUE,
  "GRTS",
  200
)
createDistMat(first.grts, "preds", T, T)
# if you didn't run the above, then make sure to run
# first.grts <- importSSN("Year_1_GRTS.ssn", "preds")
# createDistMat(first.grts)
# before moving on.

# Record the locIDs of the chosen sites
first.fixed <- as.character(getSSNdata.frame(first.grts)$locID)
```

```r
# Splice second years' sites
second.year <- spliceSSNSites(
  first.grts, "Year_2_Potential.ssn", "sites2.shp"
) # note, this will print messages from readOGR().
second.year <- importSSN("Year_2_Potential.ssn", "preds")
second.year <- subsetSSN(
  second.year, "Year_2_Selected.ssn",
  locID %in% first.fixed
)
createDistMat(second.year, "preds", T, T)
# if you didn't run the above, then make sure to run
# second.year <- importSSN("Year_2_Selected.ssn", "preds")
# createDistMat(second.year, "preds", T, T)
```

We now have a starting design. By the end of the second year of sampling, where we are now, we have collected 400 DO samples across 200 unique locations. We can use these data to fit a model and to form priors about the covariance parameters in our 'true' model based on their estimates in that model.

```r
# Fit model
first.model <- glmssn(
  Sim_Values ~ order,
  second.year,
  CorModels = c("Spherical.tailup", "locID"),
  addfunccol = "AreaAFV"
)

# Form priors
# This will give a warning that the observed information matrix
# does not exist. This is fine, and is the normal behaviour of this
# function. Instead, we will use an estimate
# of the expected fisher information.
first.priors <- constructLogNormalPriors(first.model)
```

Again, there is a seemingly unnatural step here necessitated by the file structure of `SpatialStreamNetwork` objects. We previously split our sites by year. Now, we need to reintroduce the third year of potential sampling sites back into the `SpatialStreamNetwork`. We do this with the function `spliceSSNSites`, which requires the following code:

```r
# New sites for next year
second.year <- spliceSSNSites(
  second.year, "Year_3_Potential.ssn", "sites3.shp"
)
second.year <- importSSN("Year_3_Potential.ssn", "preds")
createDistMat(second.year, "preds", T, T)

# Now save data for next run.
# You would uncomment and run the following line
# if you were planning on running optimiseSSNDesign
# over our results from before. Note, the save step is
# not necessary in many cases. We have done it here because
# we had to transfer the data from a local computer, where we
# did this preprocessing, to a high performance
# computing cluster.
# save.image("Sim_Data_Year_3.Rdata")
```

At this juncture, we can begin our adaptive design. We have two years of data, a model, and priors. We do adaptive design by calling `optimiseSSNDesign`, as shown below. Note that the code below is set up to be used on the Queensland University of Technology's High Performance Computing system, which runs a linux operating system with R v. 3.5.1. We did this to save time. This code block takes between 50 and 55 hours to run on 32 CPUs.

Note that one feature of the code that is unusual for a local machine or a smaller example is that we set `n.optim = 1`. Ordinarily, this would be too low. Setting `n.optim = 5` would have been reasonable. However, to save time, we set up five separate scripts with `n.optim = 1` and set different random seeds (1, 1e6 + 1, 2e6 + 1, 3e6 + 1, and 4e6 + 1) to achieve the same result as `n.optim = 5` but in a fifth of the time.

```
# WARNING: THIS CODE TAKES APPROXIMATELY 50 HOURS TO RUN
# USING 32 CPUs.

# Load preprocessed model and priors
# You MUST set your working directory to the folder on
# your computer containing the .Rdata file and .ssn folder
# load("Sim_Data_Year_3.Rdata")

# Import SpatialStreamNetwork object
# second.year <- importSSN(
#   "Year_3_Potential.ssn",
#   "preds"
# )

# Optimise a design
# O <- optimiseSSNDesign(
#   ssn = second.year,
#   new.ssn.path = "S_Up_1.ssn",
#   glmssn = first.model,
#   n.points = 250,
#   legacy.sites = first.fixed,
#   utility.function = KOptimality,
#   prior.parameters = first.priors,
#   n.cores = 32,
#   parallelism = "osx/linux",
#   parallelism.seed = 1,
#   # the above argument changes in increments
#   # of 1e6, being set to 1, 1e6 + 1, 2e6 + 1, etc.
#   # up to 4e6 + 1
#   # this allowed us to generate five adaptive designs
#   # from five random starts
#   n.optim = 1,
#   n.draws = 500
# )

# Save the result
# Note the number at the end of the file name changes
# according to the random start (1, 2, 3, 4, 5)
# save(O, first.priors, "S_Up_Optimal_1.Rdata")
```

Getting to this point is time-consuming; this is especially true at the last step. To give some indication of

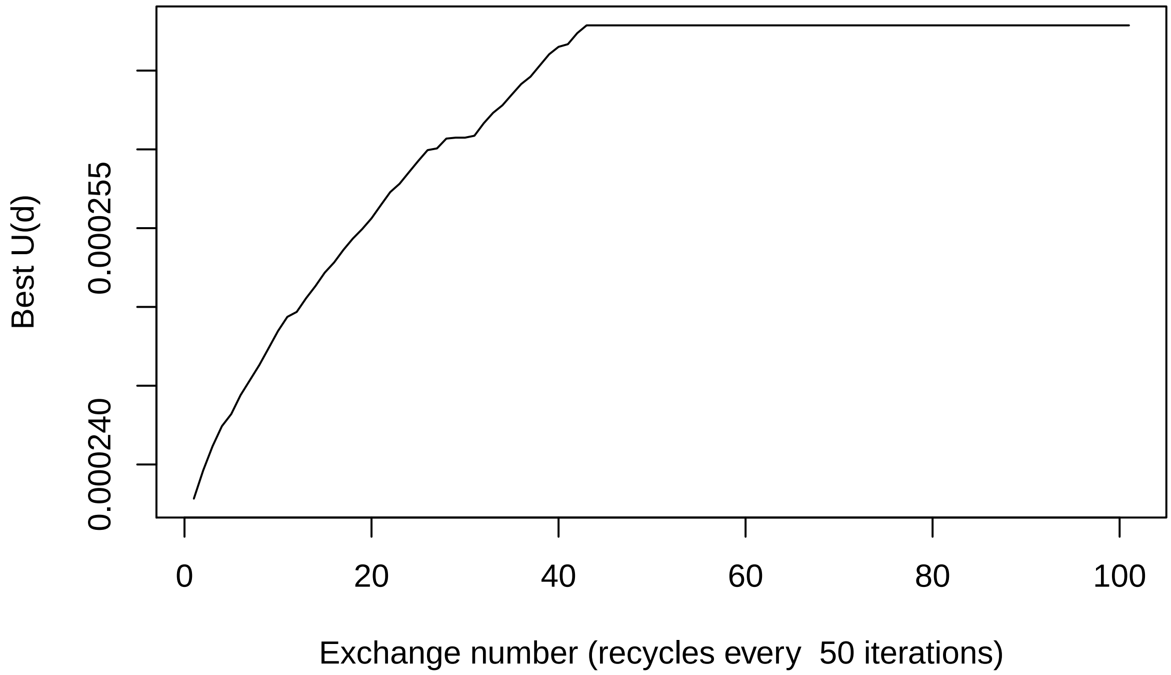

Figure 2: The trace of the Greedy Exchange Algorithm. The y-axis represents the maximum expected utility at each iteration in the algorithm.

the results, however, we load pre-processed data. Note we are loading the file `S_Up_3.Rdata` because the adaptive design found using `parallelism.seed = 2e6 + 1` produced the best result of our five optimisation runs. We load the data with

```
load("S_Up_Optimal_3.Rdata")
```

We can plot diagnostics for the optimisation algorithm, such as the trace plot of the maximum expected utility.

```
plot(O)
```

We can also plot the adaptive design, indicating which sites have been added to the design.

```
# Data frame for the adaptive design observed sites
adaptive <- getSSNdata.frame(O$ssn.new)
# New variable to code for whether the sites are legacy sites
# or were freshly added
adaptive$New <- with(adaptive, 1 * (!locID %in% O$legacy.sites))
# Return data frame to the SpatialStreamNetwork object
O$ssn.new <- putSSNdata.frame(adaptive, O$ssn.new)
# Plot
plot(O$ssn.new, "New", nclasses = 2, breaktype = "even")
```

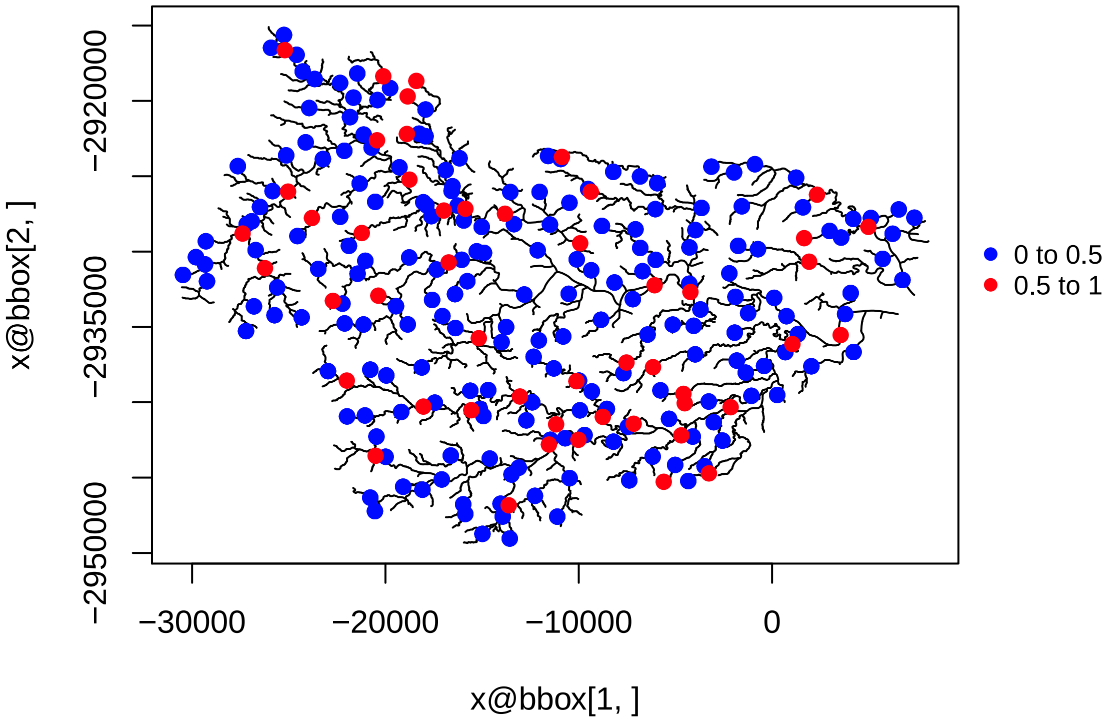

Figure 3: The adaptive design at the second step.

At this stage, we have found the adaptive design for the third year of sampling. The next task is to find the adaptive design in the fourth year of sampling. However, before this can occur, we need to

1. Refit our true model to the updated dataset (which represents the situation where we find the adaptive design, and sample from it).
2. Update our priors based on our new estimates from the updated model.
3. Update our list of legacy sites.

We do this using the following code:

```
# Read in the best adaptive design out of 5
ssn <- importSSN("./S_Up_3.ssn", "preds")
createDistMat(ssn, "preds", T, T)

# fit model to SSN
third.model <- glmssn(
  Sim_Values ~ order, ssn,
  CorModels = c("Spherical.tailup", "locID"),
  addfunccol = "AreaAFV"
)

# 'update' priors
# This will throw another warning about the observed information
# matrix, but this is okay.
third.priors <- constructLogNormalPriors(third.model)

# Update fixed sites
third.fixed <- unique(as.character(getSSNdata.frame(ssn)$locID))

# now splice in fourth year of sites
ssn <- spliceSSNSites(ssn, "Year_4_Potential.ssn", "sites4.shp")
ssn <- importSSN("Year_4_Potential.ssn", "preds")

# Save image
# Run the following line of code if you intend to run the next
# codeblock down. Note, the save step is not necessary in many
# cases. We have done it here because we had to transfer the
# data from a local computer, where we did this preprocessing,
# to a high performance computing cluster.
# save.image("Sim_Data_Year_4.Rdata")
```

The only thing that remains is to find the adaptive design for the fourth year of sampling.

```
# Load the data we saved before
# Again, you MUST set your working directory to
# the folder containing these files (the .Rdata file
# and the .ssn folder).
# load("Sim_Data_Year_4.Rdata")

# Import the fourth year of data
# with potential sites
# fourth.year <- importSSN(
#   "Year_4_Potential.ssn",
```

```r
#   "preds"
# )

# Find adaptive design
# O <- optimiseSSNDesign(
#   ssn = fourth.year,
#   new.ssn.path = "S_Up_1_1.ssn",
#   glmssn = third.model,
#   n.points = 300,
#   legacy.sites = third.fixed,
#   utility.function = KOptimality,
#   prior.parameters = third.priors,
#   n.cores = 20,
#   parallelism = "osx/linux",
#   parallelism.seed = 1,
#   ## this arguments increases in increments of
#   ## 1e6 as before
#   n.optim = 1,
#   n.draws = 500
# )

# Modify the contents of the glmssn object
# to save space in .Rdata file
# third.model$ssn.object <- NULL
# third.model$estimates$V <- NULL
# third.model$estimates$Vi <- NULL
# third.model$sampinfo$REs <- NULL
# O$ssn.old <- NULL
# O$glmssn <- NULL

# Save the workspace image
# Both numbers at the end of the filename increment
# with the random start (1, 2, 3, 4, 5)
# save(O, third.fixed, third.priors, file = "S_Up_Optimal_1_1.Rdata")
```

This gives us the final state of our adaptive design. If we want to explore this further, we can import a pre-processed dataset:

```r
load("S_Up_Optimal_3_3.Rdata")
```

We use `S_Up_Optimal_3_3.Rdata` because the optimisation run with `parallelism.seed = 2e6 + 1` again produced the adaptive design with the highest expected utility.

```r
# Same plot as before
adaptive <- getSSNdata.frame(O$ssn.new)
adaptive$New <- with(adaptive, 1 * (!locID %in% O$legacy.sites))
O$ssn.new <- putSSNdata.frame(adaptive, O$ssn.new)
plot(O$ssn.new, "New", nclasses = 2, breaktype = "even")
```

All that remains is to validate our final design. This proceeds in a few steps. Firstly, we need to import a `SpatialStreamNetwork` object containing all potential sampling sites across all years of sampling.

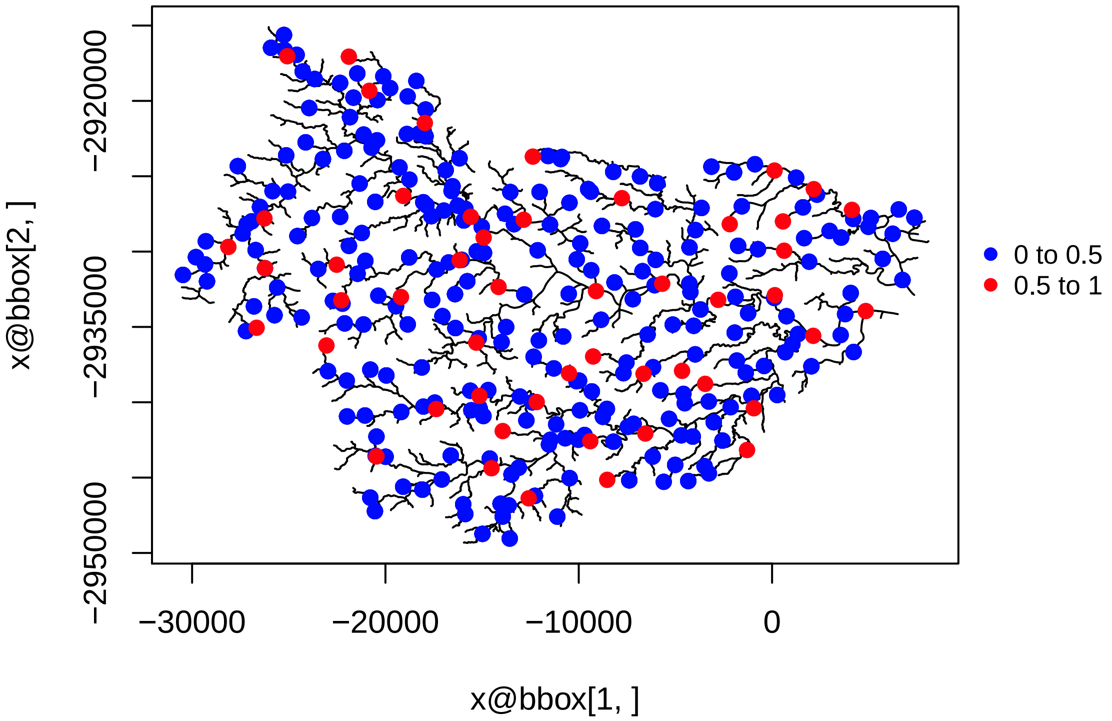

Figure 4: The adaptive design at the final step.

```r
# we need the ssn containing all POTENTIAL sampling sites for
# ALL sampling years. This will be used in evaluateFixedDesigns
total.ssn <- importSSN("./pine_river_sim_.ssn", "preds")
```

Secondly, we need to remove sites that were included in the GRTS designs we used for the first two years of sampling. This is not a step that is required in general. However, we do it because we want to compare the efficiencies of our adaptive design against some standard designs, and the adaptive design only includes the 100 sites we added between years three and four.

```r
# import adaptive design
fourth <- importSSN("./S_Up_3_3.ssn", "preds")
# import legacy sites and record them
first.two <- importSSN("Year_2_Selected.ssn")
first.fixed <- getSSNdata.frame(first.two)$locID
rm(first.two)
# record the design as a vector of pids
# but first remove the 200 fixed GRTS sites we started with
fourth.design <- with(getSSNdata.frame(fourth), pid[!locID %in% first.fixed])
opt.designs <- list(
  fourth = fourth.design
)
```

Thirdly, we set up a range of standard designs we want to validate our adaptive design against. For us, these are random and GRTS designs. We generate 20 of each to account for the range of performance we might expect to see because random and GRTS designs are stochastic.

```r
# Set a seed for reproducibility
set.seed(123456789)

# Construct some GRTS designs
# Set up an empty vector to store designs
grts.designs <- vector("list", 20)
for(i in 1:20){
  # Use this function to create temporally evolving
  # GRTS designs, using the 'master-sample' approach
  grts.designs[[i]] <- evolveGRTSOverTime(
    total.ssn,
    c(0, 0, 50, 50),
    "Year"
  )$Period_3$by.pid
}
names(grts.designs) <- rep("GRTS", 20)

# Random designs
# Set up an empty vector as before
rand.designs <- vector("list", 20)
for(i in 1:20){
  # Use this function to build up a random design
  # over time.
  rand.designs[[i]] <- evolveRandOverTime(
    total.ssn,
    c(0, 0, 50, 50),
    "Year"
```

```r
  )$Period_3$by.pid
}
names(rand.designs) <- rep("Rand", 20)
# Combine our lists of data
designs <- append(opt.designs, grts.designs)
designs <- append(designs, rand.designs)
```

Finally, we benchmark our adaptive design against the GRTS and random designs. We do two sets of benchmarking. The first set of benchmarking compares the efficiencies of the designs under the true values of the covariance parameters. THe second set of benchmarking compares the efficiencies of the designs under the last formed set of priors for the covariance parameters. In both cases, we expect the adaptive design to outperform the random and GRTS designs.

```r
# Benchmark our adpative designs aginst the GRTS and
# random designs

# compare designs under last best estimate of parameters
# warning: this can take an hour
efficiencies.emp <- evaluateFixedDesigns(
  total.ssn,
  third.model,
  designs,
  "pid",
  KOptimality,
  third.priors,
  1000
)

# save designs and results in .Rdata file
# save(designs, efficiencies.emp, file = "benchmarked.Rdata")
```

Running the above code can take 40-60 minutes, so we will load pre-processed data.

```r
load("benchmarked.Rdata")
```

The first six rows of the `data.frame` object called `efficiencies.emp` look like this:

```r
head(efficiencies.emp)
#>       ID Size Expected utility Efficiency Efficiency_Unlogged
#> 1 fourth  150     0.0003416538  1.0000000           1.0000000
#> 2   GRTS  150     0.0002837895  0.8306348           0.9999421
#> 3   GRTS  150     0.0002543829  0.7445634           0.9999127
#> 4   GRTS  150     0.0002849684  0.8340854           0.9999433
#> 5   GRTS  150     0.0002863867  0.8382367           0.9999447
#> 6   GRTS  150     0.0002786942  0.8157211           0.9999370
```

The last six rows look like this:

```r
tail(efficiencies.emp)
#>      ID Size Expected utility Efficiency Efficiency_Unlogged
#> 36 Rand  150     0.0002773608  0.8118184           0.9999357
#> 37 Rand  150     0.0002817689  0.8247207           0.9999401
```

```
#> 38 Rand  150     0.0002781254  0.8140564          0.9999365
#> 39 Rand  150     0.0002750161  0.8049556          0.9999334
#> 40 Rand  150     0.0002840155  0.8312962          0.9999424
#> 41 Rand  150     0.0002639778  0.7726472          0.9999223
```

The column `ID` represents an identifier given to the design. These are the names of the list of designs provided to `evaluateFixedDesigns` by the user but if these are null then the `ID` column will simply contain unique numerical identifiers. The `Size` column is the number of samples in the design. If the argument `list.of = "pid"` then `Size` is the number of samples across all sites across all years. If `list.of = "locID"` then `Size` is the number of unique sampling locations. The `Expected utility` column contains the expected utility for each design, and the two `Efficiency` columns are the efficiencies of the designs relative to the design with the highest expected utility. Note that `Efficiency` is calculated as a direct ratio, i.e. $U(d_i)/U(d^*)$ where d^* is the best design. In contrast, `Efficiency_Unlogged` is calculated as a ratio on the log-scale. That is, it is calculated as $\exp\{U(d_i) - U(d^*)\}$. This method is correct when $U(d_i)$ and $U(d^*)$ are log-scale expected utilities.

We can plot the results in whatever way seems appropriate. In this instance we used a boxplot of the performances of the GRTS and random designs, with a dashed line to indicate the performance of the adaptive design. The expected utility is the inverse sum of the kriging variances across our 900 prediction sites, so we made boxplots of the sum of the kriging variances for each of the design types.

```
par(mai = c(1, 0.5, 0.1, 0.1))
boxplot(
  # Note that 1/Expected utility is the sum of the
  # kriging variances
  1/`Expected utility` ~ ID,
  # Drop first row because this is the adaptive design
  efficiencies.emp[-1, ],
  ylim = c(2800, 4000),
  xlab = expression(Sum~of~kriging~variances~U(d)^-1),
  horizontal = T
)
abline(
  v = 1/efficiencies.emp$`Expected utility`[1],
  col = "red",
  lty = 2
)
```

These results indicate that random and GRTS designs perform to a similar level but that the adaptive design is more efficient. That is, we can make more accurate predictions of DO across the stream network using fewer observations when we use the adaptive design.

Summary

The `SSNdesign` package is designed to solve optimal and adaptive design problems on stream networks. The package contains functions for preprocessing stream network data and functions for finding designs for stream network data. The key functions are `drawStreamNetworkSamples`, which allows users to construct stream network designs based on standard spatial sampling schemes such as GRTS, and `optimiseSSNdesign`, which is the main workhorse function that can be used to find optimal and adaptive designs. We hope `SSNdesign` will prove a useful tool for aquatic scientists and managers.

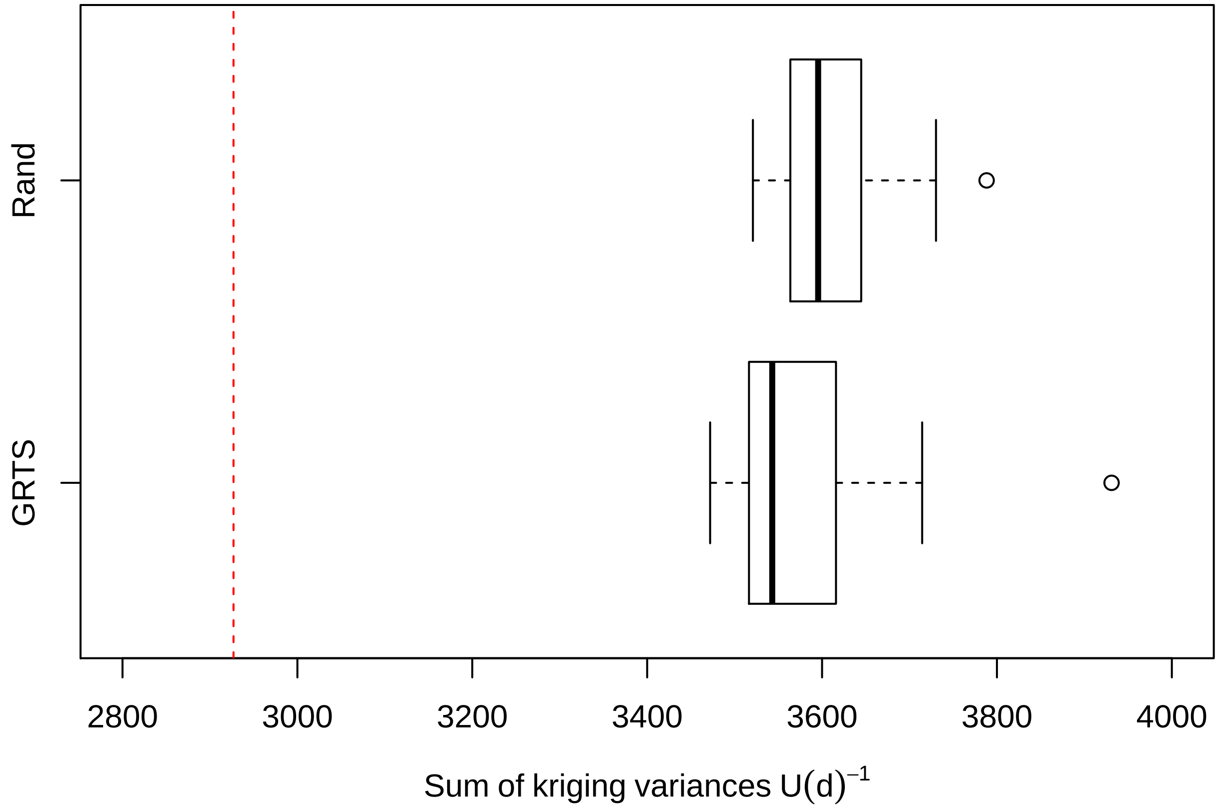

Figure 5: Sum of the kriging variances from random and GRTS designs (plotted as boxplots) compared to the sum of the kriging variances from the adaptive design (plotted as the dashed red line). A smaller sum of kriging variances indicates less uncertainty in the predictions from a model.
