# Supplementary material for: SSNdesign—An R package for pseudo-Bayesian optimal and adaptive sampling designs on stream networks
Source: PLoS One. 2020 Sep 22;15(9):e0238422. doi: 10.1371/journal.pone.0238422 (PMC7508409; doi:10.1371/journal.pone.0238422)
Supplement: Attachment — Submitted filename: Review_PONE-D-19-26876.pdf [file pone.0238422.s004.pdf]

Review of PONE-D-19-26876: SSNdesign – an R package for pseudo-Bayesian optimal and adaptive sampling designs on stream networks

Overview

The authors present a new R package- SSNdesign- to aid in solving optimal and adaptive design problems specifically for spatial stream network models (SSNMs). Due to the high cost of monitoring programs, both in terms of budget and time, it is of great importance to select sampling locations that yield as much information as possible. The first half of the manuscript lays out the R package itself and the computational details of both optimal and adaptive design techniques. The second half focuses on the application of the R package to two empirical case studies. The first study focuses on decreasing an existing monitoring network (a common design problem that comes up during long term monitoring projects), while the second study focuses on establishing a new monitoring network in a previously unsampled watershed.

First I have general thoughts about the inclusion of the case studies. In a software paper, I would expect case studies to fall under one of the following scenarios (to betterment or detriment)

1. This is a new dataset and the underlying methodology in the software is new
2. This is a new dataset and the underlying methodology in the software is not new
3. This is not a new dataset and the underlying methodology in the software is new
4. This is not a new dataset and the underlying methodology in the software is not new

Case study 1

(1) In this paper, case study one (Lake Eacham) is based on an existing dataset (i.e. Falk 2014 uses this dataset as well) and the underlying methodology used for the study is not new (optimal experimental design was used in Falk 2014 and Som 2014). I think it's important to state this clearly and cite the above-mentioned prior work.

(2) Furthermore, the case study is currently presented like a vignette (an application of the SSNdesign package to real data), but doesn't make clear how it is relevant in demonstrating the contribution of the SSNdesign package. Given that both the dataset and the methodology in this case study were already presented in prior work, the majority of content of the case study is better suited for an R package vignette or as supplementary material. Instead, I think the paper would be better served by giving (i) a more concise overview of the case study using SSNdesign, with emphasis on how the software improves the ability to do such analysis, and (ii) a discussion of how the SSNdesign package better addresses the experimental design problem compared with alternatively available software like spsurvey or geospt.

Case study 2

(1) Case study two (Pine River) is a new dataset (synthetically simulated) and the underlying methodology used for the study is new (the authors state that adaptive experimental design problems have not been used under the SSNM framework). This is a case study that supports the motivations of this paper.

In my experience, this R package will be an invaluable tool to researchers and managers in the freshwater monitoring field. I recommend this manuscript be published pending minor revisions. I look forward to reading a revised version of the manuscript.

Questions and Conceptual Comments

1. Abstract Line 17 “and so effective and efficient survey designs…”: delete “and” (double conjunction).

2. Abstract Lines 21 - 23 “Thus, unique challenges of geostatistics and…”: This sentence doesn’t feel supported by the sentences which precede it. This sentence suggests that the unique challenges of geostatistics on stream networks motivated the development of SSNdesign. But, it would be more accurate to say that these challenges motivate the development of the methodology implemented in SSNdesign. A better motivation for an R package like SSNdesign would be the lack of available software that provides access to, or application of, these methods. For example, a sentence summarizing the lack experimental design R packages which account for the unique SSN structure would be stronger motivation (i.e. see 5th paragraph, lines 94 - 109 of the introduction).

3. Lines 58 - 61: Here the authors have demonstrated the increased use of SSNMs in the literature. The end of this paragraph would be a good place to cite other papers which empirically looked at experimental design utilizing SSNMs. I think it would strengthen the paper’s motivation and further highlight the research needs of an R package which tackles these questions. For example Marsha et al, 2018, Monitoring riverine thermal regimes on stream networks: Insights into spatial sampling design from the Snoqualmie River, WA, Ecological Indicators 84: 11-26. Some of the takeaways from these papers align with those learned from the case studies you have included.

4. Line 67 “Utility functions are mathematical…”: This sentence feels out of place here. Suggest moving it to line 70 before “A variety of utility functions are available…”

5. Line 81 “which measure the suitability of an experimental design for some purpose”: The authors already defined a utility function in the previous paragraph. I suggest deleting this and continue with “often depend on…”

6. Line 87 “In this paper and in SSNdesign…”: Upon first reading I don’t understand the separation between “this paper” and “SSNdesign.” By this paper do you mean application to the case studies? Perhaps make this clearer.

7. Figure 1:
    (1) It is not clear where the reader should be starting with this flowchart. My experience with using SSNMs in R indicate to me how to interpret this flowchart, but I can see how a reader who is new to working with SSNMs in R could be

confused. A flow chart which is left justified (or column aligned) and which then flows in parallel would be clearer (like Figure 2).

(2) When interpreting this flowchart it is not clear if the two ".ssn folder" objects are the same object fundamentally. If they are, then the reader wonders why one is required to be run through the **importSSN** function before **SimulateOnSSN**, while the other can go straight to **SimulateOnSSN**. My interpretation is that the bottom path "**createSSN** → .ssn folder → **SimulateOnSSN**" represents a continued R session (the scenario where the user starts with nothing); while the other path represents a new R session where the user starts with a ".ssn folder" object. If this interpretation is correct, I would suggest splitting this figure into two sections (one right and one left; or one top and one bottom) illustrating the two scenarios.

8. The *Expected utility estimation and maximisation* section: In general these two paragraphs are concise and very well explained.

9. The *Utility functions for optimal and adaptive experimental designs* section:
    (1) Reading about the adaptive design technique in SSNdesign, I see that maximizing $U(d \mid d_{0:t-1}, y_{0:t-1})$ depends upon d_0 (an initial model design). This naturally makes the reader wonder the sensitivity of the initial model chosen. Did the authors look at how the choice of d_0 affects future iterations, or even the final outcome, in their adaptive design? Or are there any other studies that looked at this that can be cited?
    (2) I see where the authors explicitly discuss solving adaptive design problems (paragraph 2), but where are optimal design problems discussed in this section?

10. Line 195 "Space-filling designs are designs which…": delete "are designs which, ideally" so it reads "Space-filling designs contain roughly…"

11. Line 201 "Here we used an algorithm…": change "used" to "use"

12. Line 219 "Users may also define…": change to "Users may also define their own utility functions since the **optimiseSSNDesign** function has the flexibility to…" It doesn't make sense to use a function to define another function.

13. Line 258 "Field data collection can be expensive and so existing…": delete "and" (double conjunction).

14. Lines 271 - 275: Just a formatting note to capitalize "Identify" and "Set" as the beginning of each list point.

15. Line 274 "Set priors that specify what the…": I suggest changing this sentence as follows, "Set priors that specify the likely relationship between covariates and the response based on expert opinion and/or a literature review." This feels more concise and clear.

16. Line 281: This is a previously published dataset that should be cited.

17. Figure 4: This is a good figure which effectively illustrates the author's conclusions from case study 1.
    (1) I suggest the two plots use the same y-axis scale (0.0 – 1.0). It could be misleading to the reader otherwise.
    (2) The feature in this figure that stands out to me the most is the lack of variability in efficiency under the GRTS designs using the CPD-optimal utility function (a) and the larger variability under the GRTS designs using the K-optimal utility function(b). Do the authors have any ideas about why that would be?

18. Line 382 "Compared to other packages…": change to "Compared with other packages…" Typical convention is that "compared to" is used for discussing similarities between fundamentally different objects while "compared with" is for discussing differences between two fundamentally similar objects.
