# Supplementary material for: SSNdesign—An R package for pseudo-Bayesian optimal and adaptive sampling designs on stream networks
Source: PLoS One. 2020 Sep 22;15(9):e0238422. doi: 10.1371/journal.pone.0238422 (PMC7508409; doi:10.1371/journal.pone.0238422)
Supplement: S1 Appendix — (PDF) [file pone.0238422.s001.pdf]

S1 Appendix: Background on optimal and adaptive pseudo-Bayesian design

Alan R. Pearse, James M. McGree, Nicholas A. Som, Catherine Leigh, Paul Maxwell, Jay M. Ver Hoef, Erin E. Peterson

2020-07-08

Notation for geostatistical models and experimental designs

Geostatistical models (1), specifically, spatial stream network models (2), are a fundamental aspect of our work in experimental design for stream networks. Our utility functions which enable optimisation of experimental designs are derived from various matrices and theoretical aspects of these models. Here, we provide definitions of fundamental statistical elements of these models (Table 1).

Moving average autocovariance models for stream networks

As geostatistical domains, stream networks exhibit greater constraints than the conventional domain of 2D Euclidean space. The unique branching nature of stream networks and the unidirectional flow of water within these branches necessitates the development and use of special spatial covariance functions. This is what distinguishes stream network models and the design of experiments for stream network models from similar problems in geostatistics.

Consider a stream network as a set of lines (stream segments) that branch upstream from the most downstream segment on the network (outlet segment) to the most upstream segments on the network (headwaters). We assume that the branching is binary (i.e. three or more segments never branch upstream from the same confluence). Observations are represented by points on the network, which have two coordinate systems (3); one is the usual two-dimensional coordinate system, and the other is based on the network topology (i.e. branching structure and connectivity of segments). Note that separation distance between two locations along the network (e.g. stream distance) is the shortest distance between them when movement is confined to the network (4). If water flows from an upstream location to a downstream location, we refer to these

Table 1: Definitions of statistical elements of spatial stream network models

**Quantity**	**Definition**	**Additional notes**
y	Vector of the observed data	Only the values observed from the dependent variable of interest are referred to as 'the data'.
X	The design matrix.	The first column is a column of 1s for models containing an intercept term. The other columns relate to covariate values at the observed sites.
θ	Vector of the model parameters	The vector θ is a general vector of parameters for a geostatistical model, including both the covariance parameters and fixed effects parameters. Note, however, that in many contexts it refers only to the covariance parameters. A note will be made whenever this vector also includes the fixed effects parameters.
β	Vector of the fixed effects parameters	
$D(s_i, s_j)$	Distance between the sites s_i and s_j	Distance can be measured in a number of ways. Euclidean distance is the most common measurement for classical geostatistics; however, stream networks can also use hydrological distance.
Z	Distance matrix between all sites in a design	This is a summetric matrix where each element Z_{ij} is some distance between sites x_i and x_j. When $i = j$, $Z_{ij} = 0$ by necessity.
Σ	Covariance matrix on the data	The elements Σ_{ij} are covariances between pairs of sites x_i and x_j, depending only on $D(s_i, s_j)$ and θ in a covariance function. $\Sigma(\theta)$ represents the case when θ is assumed to be known; $\Sigma(\hat{\theta})$, the case when $\hat{\theta}$ must be estimated from the data.
$\hat{\beta}_{gls}$	Vector of the esimated fixed effects parameters	The estimator is the generalised least squares (GLS) estimator, which has the form $\hat{\beta}_{gls} = (X^T\Sigma^{-1}X)^{-1}(X^T\Sigma^{-1}y)$ This is a best linear unbiased estimator (BLUE).
$Var(\hat{\beta}_{gls})$	Covariance matrix of the fixed effects	This matrix summarises the uncertainty and interdependences in the estimates of $\hat{\beta}_{gls}$. It is defined as $Var(\hat{\beta}_{gls}) = (X^T\Sigma^{-1}X)^{-1}$.
$s_z \in S$	Prediction sites	The set of all prediction sites is written as S.
X_s	Design matrix for the prediction sites	The first column is a column of 1s for models containing an intercept term. The other columns contain values of the covariates recorded at individual prediction sites.
V	Covariance matrix on the prediction sites	This is found with the pairwise distances between prediction sites and θ in the covariance function.
C	Covariance matrix between the observed and prediction sites	This is found with the pairwise distances between observed and prediction sites and θ in the covariance function.
$\hat{y}(s_z)$	Prediction at a prediction site	The predicted value of the response variable at the prediction site s_z. This is determined using the best linear unbiased predictor (BLUP; Cressie, 1993).
$Var(\hat{y}(s_z))$	Kriging variance at prediction sites	Prediction uncertainty is expressed via kriging variance, which in the universal kriging system is expressed as the diagonal elements of: $V - C^T\Sigma^{-1}C + (X_s - X^T\Sigma^{-1}C)^T(X^T\Sigma^{-1}X)^{-1}(X_s - X^T\Sigma^{-1}C)$.

locations as flow-connected, and we refer to two locations within the same network not connected by flowing water as flow-unconnected.

Models for stream networks, based on moving average constructions, were initially described by Ver Hoef et al. (5) and Cressie et al. (6). The models summarized in Hoef and Peterson (2) extend this work and use a spatial moving-average approach to construct Gaussian random fields based on the network topology, rather than the usual two-dimensional coordinate system commonly used in geostatistics. These approaches yield random processes that are similar to typical geostatistical models; they can be described by a mean function that depends on the location within the network, and a second-order stationary covariance function. Traditional covariance functions parameterise the dependence between observations in terms of the Euclidean distance separating two locations, but this is less straightforward in the context of stream networks. Stream network covariance functions and the distance metrics they use may depend on flow connectivity. Details on these covariance functions are provided below.

Using the moving average constructions, if a moving average function is non-zero only upstream of a location, it is called a "tail-up" model. The function must split at confluences as it goes upstream to maintain stationarity of variances, so some weighting of segments must occur. If a moving average function is non-zero only downstream of a location, it is called a "tail-down" model. Consider two pairs of sites that have the same stream distance between them, but one pair is flow-connected, and the other pair is flow-unconnected; in general the amount of autocorrelation will be different between them. Let r_i and s_j denote two locations on a stream network, and let h be the stream distance between them. Then the following models have been developed to describe different forms of covariance of the response at locations r_i and s_j.

The moving average construction for tail-up models, as described by Ver Hoef et al. (5), is

$$C_u(r_i, s_j|\theta_u) = \begin{cases} \pi_{i,j} C_t(h|\theta_u) & \text{if } r_i \text{ and } s_j \text{ are flow-connected,} \\ 0 & \text{if } r_i \text{ and } s_j \text{ are flow-unconnected,} \end{cases} \quad (1)$$

where $C_u(r_i, s_j|\theta_u)$ is the spatial autocovariance between r_i and s_j, u denotes a tail-up model, θ_u is the vector of covariance function parameters, $C_t(h|\theta_u)$ is the value of a covariance function based on h and θ_u, and a selected covariance model (e.g. exponential), and $\pi_{i,j}$ are weights to account for the branching characteristics of the stream and maintain variance stationarity. The weights reflect the relative shared flow among locations, and more details can be found in Ver Hoef and Peterson (2), including ways to create an additive function from values associated with stream segments, such as flow volume, a proxy for flow volume (e.g. basin area), or any other ecologically relevant variable.

For this introduction we focus on the exponential stream-network covariance function because its geostatistical counterpart is frequently applied by practitioners, but there are many other useful covariance functions, and we encourage interested readers to explore them among the stream-network covariance model citations. For the exponential stream-network covariance function, $C_t(h|\theta_u)$ has the following form (5):

$$C_t(h|\theta_u) = \sigma_u^2 \exp(-3h/\alpha_u), \tag{2}$$

where $\sigma_u^2 > 0$ is an overall variance parameter (also known as the partial sill), $\alpha_u > 0$ is the range parameter, and $\theta_u = (\sigma_u^2, \alpha_u)'$. Via Equation (1), spatial autocorrelation is only permitted between flow-connected locations in the tail-up model.

For tail-down models, spatial autocorrelation is permitted between both flow-connected and flow-unconnected locations, but we generally distinguish between the two cases. When two sites are flow-unconnected, there will always be at least one common confluence (i.e. a downstream confluence that receives water from each of the two upstream sites). Let b denote the longer of the two distances to the closest common downstream confluence, and a denote the shorter of the two distances. If two sites are flow-connected, again use h to denote their stream distance. Again, the only tail-down model we consider is the exponential, defined as follows:

$$C_d(a, b, h|\theta_d) = \begin{cases} \sigma_d^2 \exp(-3h/\alpha_d) & \text{if flow-connected,} \\ \sigma_d^2 \exp(-3(a+b)/\alpha_d) & \text{if flow-unconnected,} \end{cases} \tag{3}$$

where $C_d(a, b, h|\theta_d)$ is the spatial autocovariance between r_i and s_j, $\sigma_d^2 > 0$ is an overall variance parameter, $\alpha_d > 0$ is the range parameter, $\theta_d = (\sigma_d^2, \alpha_d)'$, and d denotes a tail-down model. We note, for the exponential model, that the flow-connected and flow-unconnected models are equivalent, and stress this is a unique property of the exponential form of tail-down covariance models (7). A full development and more detail regarding the suite of stream-network moving-average models can be found in Ver Hoef and Peterson (2).

A mixed linear model combining tail-up and tail-down components is

$$Y = X\beta + z_u + z_d + \epsilon, \tag{4}$$

where Y is the vector of random variables for an observable stream attribute at sampled locations, X is a design matrix with full column rank for the fixed effects, β contains fixed effects parameters, z_u contains

spatially-autocorrelated random variables with a tail-up autocovariance (e.g. Equation (2)), with $\mathrm{var}(z_u) = \sigma_u^2 R(\alpha_u)$ and $R(\alpha_u)$ is a correlation matrix that depends on the range parameter α_u; z_d contains spatially-autocorrelated random variables with a tail-down autocovariance (e.g. Equation (3)) such that $\mathrm{var}(z_d) = \sigma_d^2 R(\alpha_d)$; and ϵ contains independent random variables with $\mathrm{var}(\epsilon) = \sigma_0^2 I$. When used for spatial prediction, this model is referred to as “universal” kriging (8), with “ordinary”" kriging being the special case where the design matrix X is a single column of ones (1). This yields a covariance matrix of the form

$$\mathrm{var}(Y) = \Sigma = \sigma_u^2 R(\alpha_u) + \sigma_d^2 R(\alpha_d) + \sigma_0^2 I. \tag{5}$$

The expected utility

In Bayesian and pseudo-Bayesian experimental design, an optimal design d^* is found by maximising an expected utility function $U(d)$ through the choice of design d from a set of possible designs D. The expected utility function is specified to capture the goal of data collection, such as precise estimation of model parameters and accurate prediction of a response at unobserved locations.

To define the expected utility, we first define the utility function denoted as $U(d, \theta, y)$ which depends on a vector of parameters from a geostatistical model $\theta \sim p(\theta)$ and the data we expect to observe under that model $y \sim p(y|\theta, d)$. Note, however, that many pseudo-Bayesian utility functions do not depend on y and so in many cases the utility function can simply be written $U(d, \theta)$ (9). The utility function then specifies the aim of data collection. For example, one may be interested in the precise estimation of the parameters. In this case, we could define our utility function as the negative sum of the variances for each parameter estimate. We take the negative sum because we generally define utility functions such that they should be maximised. As the notation suggests, such a quantity depends on the design d, the data y and on θ through the likelihood of y. However, in reality, we do not know what data will be observed, and hence we cannot evaluate $U(d, \theta, y)$ directly to design experiments. Instead, we use prior information about θ and y to capture their joint distribution, and integrate $U(d, \theta, y)$ over this uncertainty. This leads to the following definition of the expected utility (10):

$$U(d) = \int_\theta \int_y U(d, \theta, y) p(y|\theta, d) p(\theta) \; dy \; d\theta. \tag{6}$$

This integral is slightly modified for pseudo-Bayesian utility functions $U(d, \theta)$ that do not depend on y. For these utility functions, the integral which gives the expected utility is simplified to an integral over the

parameters θ such that

$$U(d) = \int_{\theta} U(d,\theta)p(\theta)\ d\theta. \tag{7}$$

Unfortunately, the above integrals are generally analytically intractable for most applications, meaning that they have no closed form solution. In practice, this is inconvenient but we can still approximate the expected utility. Monte Carlo integration (Algorithm 1) is commonly used for this purpose (11). For a utility function $U(d, \theta, y)$, Monte Carlo integration requires taking M draws from the prior $\theta^{(m)} \sim p(\theta)$ and then the likelihood $y^{(m)} \sim p(y|\theta^{(m)}, d)$. For each m, the utility function is evaluated for $\theta^{(m)}, y^{(m)}$ to give $U(d, \theta^{(m)}, y^{(m)})$. For a utility function $U(d, \theta)$, the process is the same but we ignore $y^{(m)} \sim p(y|\theta^{(m)}, d)$. The values of the utilities are then averaged such that $U(d) \approx \sum_{m=1}^{M} U(d, \theta^{(m)}, y^{(m)})/M$ or $U(d) \approx \sum_{m=1}^{M} U(d, \theta^{(m)})/M$ depending on whether the utility function depends on the parameters and the data or the parameters only. In order to accurately estimate $U(d)$, we need large M, which usually means $M \geq 500$.

Algorithm 1 Algorithm for estimating the expected utility $U(d)$ by Monte Carlo integration

1: Specify $U(d, \theta, y)$ or $U(d, \theta)$ as appropriate.
2: Specify a prior $p(\theta)$ and, if necessary, the likelihood $p(y|\theta, d)$.
3: Set M to be the total number of Monte Carlo draws to be used for approximating $U(d)$.
4: **for** $m = 1 : M$ # each Monte Carlo draw # **do**
5:     Take $\theta^{(m)} \sim p(\theta)$.
6:     Take $y^{(m)} \sim p(y|\theta^{(m)}, d)$ if required.
7:     Evaluate $U(d, \theta^{(m)}, y^{(m)})$ or $U(d, \theta^{(m)})$.
8: **end for**
9: Evaluate $U(d) \approx \sum_{m=1}^{M} U(d, \theta^{(m)}, y^{(m)})/M$ or $U(d) \approx \sum_{m=1}^{M} U(d, \theta^{(m)})/M$ as appropriate.

Searching for an optimal design

In this section, we outline how we maximise the expected utility function over a set of possible designs (Algorithm 2). The use of optimisation algorithms such as exchange algorithms (12) is necessary because many design problems are impossible to solve analytically and are too large to efficiently solve numerically with a computer under a brute-force search scheme. If one wants to find an optimal design of size n and there are N sites to choose from, then the optimal design d^* will exist somewhere among N choose n potential solutions. This number is exceedingly large for all but the smallest values of N. Therefore, an optimisation algorithm is used to greatly reduce the costs associated with the search for d^* (12).

In this work, we use a greedy exchange algorithm (Algorithm 2) to locate optimal designs (9,13). The

greedy exchange algorithm works by optimising the choice of each of n sites one-by-one. Initially, a random design with n sites is proposed and becomes $d_0 = \{s_1, s_2, ..., s_n\}$ (the initial design) and d^* (the design which currently has the highest value of $U(d)$). From this point, we begin the coordinate exchange. Note that there are $N - n$ candidate points not currently in d^*. The first site in d_0 (s_1) is then swapped out for each of the $N - n$ candidate sites. The expected utilities of the resulting designs are recorded. If any designs have an expected utility larger than $U(d^*)$, the design with the highest expected utility replaces d^*. Then we update our pool of candidate sites, and we begin to exchange the next site. Otherwise, the design reverts to d^* and the next site in the design is exchanged for each candidate site. This process continues until we have exchanged all n sites. If d^* changed at any point in this process (i.e. if we see any improvement in the design), we repeat the sequence of exchanges. This continues until we finally observe no improvement in the expected utility of d^*. We then exit the algorithm. The algorithm is called the greedy exchange algorithm because it only accepts improvements in the design and stops when no further improvement can be achieved.

Algorithm 2 The greedy exchange algorithm

Set K, the number of searches from random starts. This is to mitigate against becoming trapped in local maxima.
for $k = 1 : K$ **do**
  Initialise d_0 as a randomly selected design with n of N points.
  Set $d^* = d_0$
  Evaluate $U_0 = U(d_0)$ to initialise the search for designs
  Store $U^* = U_0$; the expected utility of the global 'best design'.
  Temporarily store $U^*_{ij} = U^* + \epsilon$ (the expected utility of the 'best-within-search' design) for small ϵ. (This is simply to force the while loop to iterate at least once.)
  Initialise $U_{ij} = \emptyset$. U_{ij} will be used to store the expected utilities for designs evaluated during the following search.
  while $U^*_{ij} > U^*$ # until there is no improvement # **do**
    Update $U^* = U^*_{ij}$
    for $i = 1 : n$ # each point currently in the design # **do**
      Find the $N - n$ points not in d^*
      for $j = 1 : (N - n)$ # each point not in the design # **do**
        Form d_{ij} by swapping out the ith point in the design for the jth point not in the design
        Evaluate $U = U(d_{ij})$
        Define $U_{ij} = U_{ij} \cup U$
      end for
      if $\max(U_{ij}) > U^*_{ij}$ **then**
        Update $d^* = \text{argmax}_{d \in D} U(d_{ij})$
        Update $U^*_{ij} = U(d^*)$
      else
        Keep the previous d^* and U^*_{ij}.
      end if
    end for
  end while
end for

The greedy exchange algorithm can be analysed to yield an approximate expression for the run-time. We first

assume that estimating $U(d)$ is the most time-consuming part of the algorithm and that any intermediate storage operations and data manipulation between evaluations of $U(d)$ are inconsequential. Let us assume that it takes S seconds to evaluate $U(d)$. The number of expected utilities that are calculated in the greedy exchange algorithm each time an optimal design is found (for each of K iterations, in Algorithm 2) is stochastic. However, the stochasticity is due to only a single step (the condition at Line 9, Algorithm 2) and the number of times the expected utility must be estimated is otherwise well-constrained. There are L iterations, and L is random. In every iteration of the greedy exchange, there are $n \times (N - n)$ designs to be evaluated. This process is repeated K times from K random starts, so the number of times the expected utility is estimated is $K \times L \times n \times (N - n) + K$. The additional K evaluations are from the random starts, which must be evaluated. However, K extra evaluations of $U(d)$ are unlikely to be of any consequence for calculating expected run-time, so we discard them. Altogether, the expected run-time for the greedy exchange algorithm is $K \times L \times n \times (N - n) \times S$ seconds. However, some utility functions have large S and therefore expected runtimes may still be large. Therefore, we use parallel computing to further reduce the total runtime. Let C be the number of CPUs across which the greedy exchange algorithm is to be run. Then the expected runtime reduces to approximately $(K \times L \times n \times (N - n) \times S)/C$ seconds.

Utility functions for static optimal design

In this section, we set out detailed notation and explanations of our utility functions. In our package, we have provided an off-the-shelf utility function for

- Precision of covariance parameter estimation
- Precision of fixed effect parameter estimation
- Precision of estimation of both covariance and fixed effect parameters
- Precision of predictions, and
- Approximately evenly-spaced sites in the stream network.

Our covariance parameter estimation utility is called CP-optimality and was used in both Falk et al. (9) and Som et al. (14). It is given by

$$U(d, \theta) = \log \det \left[I(d, \theta) \right], \tag{8}$$

where $I(d, \theta)$ is the expected Fisher information for the covariance parameters. To compute the expected

Fisher information, we use the restricted error maximum likelihood (REML) estimator (9,14). This means each element $[I_{i,j}(\theta, d)]$ is defined by

$$I_{i,j}(\theta, d) = \frac{1}{2}\text{tr}\left(\frac{\partial \Sigma}{\partial \theta_i} P \frac{\partial \Sigma}{\partial \theta_j} P\right), \tag{9}$$

where the matrix P in Equation 9 is defined as $P = \Sigma^{-1} - \Sigma^{-1} X \left(X^T \Sigma^{-1} X\right)^{-1} X^T \Sigma^{-1}$. This utility function works because larger values of $\det[I(d, \theta)]$ correspond to lower uncertainties on θ, as given by the elements of $I(d, \theta)$.

Our fixed effects estimation utility is called D-optimality (9,14). This utility works on the same principle as CP-optimality, though it minimises the uncertainty in a different set of parameters. Formally, the utility function is

$$U(d, \theta) = \log\det\left[I(d, \beta_{\text{gls}})\right], \tag{10}$$

where $I(d, \beta_{\text{gls}}) = Var(\beta_{\text{gls}}, d)^{-1} = X^T \Sigma^{-1} X$ is the Fisher information for the fixed effects parameters. Note that this assumes the covariance parameters are known up to a prior. For cases when there is little information about the covariance parameters and it is advantageous to estimate them from the data, we use empirical D-optimality (ED-optimality, after Som et al. (14)). In this case, the criterion is modified from Equation 10 such that

$$U(d, \theta, y) = \log\det\left[I(d, \hat{\beta}_{\text{gls}})\right], \tag{11}$$

where $I(d, \hat{\beta})$ is the observed Fisher information for the fixed effects parameters. For the empirical D-optimality utility function, the vector θ includes the fixed effects β. These are needed to generate the data y from which $\hat{\beta_{\text{gls}}}$ are estimated. Though Som et al. (14) adjust the utility function with the addition of another quantity derived from the inverse Fisher information, we do not. Their reasoning for making this adjustment was to account for changes to the sampling distributions of the fixed effects when the covariance parameters are estimated from the data. However, since we are averaging over a set of prior draws for the covariance parameters, we are in effect constructing the sampling distribution of the fixed effects through simulation.

A dual purpose utility function is also defined for improving the precision of both fixed effects and covariance parameter estimates at the same time. We call this CPD-optimality. Instead of considering the information

matrices for the fixed effects and covariance parameters separately, we consider a combination of the two as a block diagonal matrix such that

$$F = \begin{pmatrix} D & 0 \\ 0 & C \end{pmatrix}, \tag{12}$$

where $D = I(d, \beta_{\text{gls}})$ and $C = I(d, \theta)$. Again we define our utility function as the log-determinant of this matrix, which reduces to

$$U(d, \theta) = \log\left[\det(D)\det(C)\right] = \log\left[\det(D)\right] + \log\left[\det(C)\right]. \tag{13}$$

This is simply the sum of the two utility functions D- and CP-optimality.

Our prediction utility is called K-optimality, where K is for kriging. It is the inverse sum of the kriging variances defined at a set of prediction sites $s_z \in S$ for $z = 1, ..., Z$ where Z is the number of prediction sites. This utility function favours designs where the total uncertainty is small. When covariance parameters are known (15), this is

$$U(d, \theta) = \left(\sum_{s_z \in S} \text{var}\left(\hat{y}(s_z, \theta), d\right) \right)^{-1}. \tag{14}$$

We use the universal kriging system to estimate the kriging variances (1). When we need to empirically estimate the covariance parameters due to a lack of strong beliefs about them, we can use empirical kriging variances. In this situation, we get the empirical K-optimality function (EK-optimality), which is

$$U(d, \theta, y) = \left(\sum_{s_z \in S} \hat{\text{var}}\left(\hat{y}(s_z, \hat{\theta}), d\right) \right)^{-1}. \tag{15}$$

The vector θ includes the fixed effects β because they are needed to generate $y^{(m)} \sim p(y|\theta^{(m)}, d)$ in Alg. 1. Note there is a parameter estimation step in this empirical utility, so it serves the dual purpose of prediction accuracy and parameter estimation (9).

Two space-filling utilities are also provided in the package. Space-filling designs are used to construct designs with roughly equally spaced and unclustered sets of monitoring sites in space. The first space-filling utility function is the maximin utility function (16), which is

$$U(d) = \min_{i \neq j} D(s_i, s_j), \tag{16}$$

where the distance $D(s_i, s_j)$ (Table 1) can be either Euclidean or hydrological distance (2). This utility function unsurprisingly favours configurations of sites that maximise the minimum distance among any two sites. The second is the modified maximin design criterion proposed by Morris and Mitchell (17). This is

$$U(d) = -\left(\sum_{i=1}^{w} (J_i Z_i)^p\right)^{1/p}. \tag{17}$$

In this utility function, w is the number of unique non-zero distances between pairs of points in a design. The vector Z contains w distance elements sorted from smallest to largest. The vector J contains the number of times each of these distances occur in one triangle (upper or lower) of the distance matrix for a given design. The parameter p is a weighting power which determines the weighting to be given to smaller distances. As $p \to \infty$, the contribution of the smallest non-zero distance Z_1 to the utility will far outweigh the contribution of any other term in the sum and this utility will reduce to the maximin utility described earlier. Note that the value of p is arbitrary and user defined. Morris and Mitchell (17) recommend that p be set between $p \in [20, 40]$ but any $p > 1$ is viable. Compared to the maximin utility function, this utility function has the advantage of being able to incorporate information about the distances between pairs of points in the design which are larger than the minimum distance, with a view to providing a more spatially balanced design where not only is the minimum distance between points large but that larger distances also increase accordingly. As a final note on these two utility functions, it can be seen that neither depend on θ or y. Therefore, no integration is needed to obtain the expected utility.

Utility functions for adaptive design

Adaptive designs differ from optimal designs because, instead of making a single decision about where to sample within a stream network, adaptive designs involve a series of decisions about where to sample that evolve over time as new data becomes available. We use a myopic design approach in `SSNdesign`, which means that we only look one step forward in the series of design decisions we have to make and try to make the best decision for the next timestep only. This is in contrast to backward induction, which involves enumerating every possible decision we could make in the future and selecting the series of decisions that, retrospectively, should lead to the best result (18).

Adaptive designs account for the designs used and data collected at previous timesteps by modifying the expected utility function (Algorithm 3). Let t be a timestep with $t = 0, 1, 2, ..., T$ for some total number of time periods T. At time period t, a total of $t - 1$ design decisions and datasets have been collected. In adaptive design, we leverage this information to improve our design. Therefore, our expected utility function can be written as $U(d|d_{0:t-1}, y_{0:t-1})$. That is, the utility of any design under consideration in the current time period depends on the designs and data from all previous time periods. To avoid continually refitting models to a potentially large number of data points (i.e. data from previous timesteps), we summarise the information obtained about θ from previous timesteps through a summary statistic $O_t(d_{0:t}, y_{0:t}, \theta)$. An example of such a summary statistic that we frequently use is the observed Fisher information about θ from previous time steps. Expected utility functions can then be interpreted as evaluating the information gain that is additional to what has been previous observed. Then, within this context, expected utility functions are optimised as described in Algorithm 2 for time period t.

Algorithm 3 Algorithm for finding adaptive designs

```
Initialise d_0 and y_0
Estimate θ given y_0 and d_0 to form p(θ|d_0, y_0)
Obtain summary of model fit, e.g. O_0(d_0, y_0, θ)
for t = 1 : T do
    Find d_t = max_{d∈D} U(d|d_{0:t−1}, y_{0:t−1}) where U(d|d_{0:t−1}, y_{0:t−1}) depends on all previous design decisions
    through the statistic O_{t−1}(d_0, y_0, θ)
    Collect new data y_t in accordance with d_t. If no data collection can be performed, simulate data
    collection by generating y_t from the data-generating model (p(y|θ, d_t)), with assumed parameters θ
    and the design under consideration d_t.
    Estimate θ given y_{0:t} and d_{0:t} to form p(θ|d_{0:t}, y_{0:t})
    Update the statistic O_t(d_{0:t}, y_{0:t}, θ)
end for
```

We have included three utility functions for adaptive design in our package:

- Sequential CP-optimality, for adaptive covariance parameter estimation.
- Sequential D-optimality, for adaptive fixed effects estimation.
- Sequential ED-optimality, for adaptive fixed effects estimation with empirically estimated covariance parameters.

In sequential CP-optimality, we define $O_t(d_{0:t}, y_{0:t}, \theta)$ to be the observed Fisher information matrix for the covariance parameters from a spatial stream network model fitted over the existing design. This leads to the following definition of sequential CP-optimality:

$$U(d, \theta|d_{0:t-1}, y_{0:t-1}) = \log\det\left[I(d, \theta) + O_{t-1}(d_{0:t-1}, y_{0:t-1}, \theta)\right] \quad (18)$$

Note that, in practice, we cannot guarantee that it will always be possible to run this utility function (`sequentialCPOptimality`) because the observed Fisher information matrix for the covariance parameters is not always returned in objects of class `glmssn`.

In sequential D-optimality and ED-optimality, we define $O_t(d_{0:t}, y_{0:t}, \theta)$ to be the observed Fisher information matrix for the fixed effects. We obtain this by fitting a stream network model over the data that have been collected using the existing design. The sequential D-optimality function is effectively the same as Equation 18 where β is substituted in for θ. The sequential ED-optimality function is similar, except that it uses the observed Fisher information matrix for the fixed effects $I(d, \hat{\beta})$ instead of the expected Fisher information matrix $I(d, \beta)$ like sequential D-optimality. Therefore, the utility function is written as

$$U(d, \theta, y_t | d_{0:t-1}, y_{0:t-1}) = \log\det\left[I(d, \hat{\beta}) + O_{t-1}(d_{0:t-1}, y_{0:t-1}, \theta)\right]. \quad (19)$$

No special functions are defined as adaptive equivalents of K and EK-optimality. This is because the only appropriate quantity that might be used as $O_t(\theta)$ for K and EK-optimality is the sum or inverse sum of the kriging variances defined at the prediction sites. However, simply adding this quantity in the utility function would have no impact on the results because it would offset every calculation by the same amount. Instead, K and EK-optimality can both be used 'as-is' for adaptive designs. Each optimisation will still be conditioned on previous designs and observed data through any legacy sites in the design, as well as through the updated estimates and priors of parameters in the spatial stream network model.

References

1. Cressie N. Statistics for spatial data. Wiley, New York; 1993.

2. Ver Hoef JM, Peterson EE. A moving average approach for spatial statistical models of stream networks. Journal of the American Statistical Association. 2010;105(489).

3. Peterson EE, Ver Hoef JM, Isaak DJ, Falke JA, Fortin M-J, Jordan CE, et al. Modelling dendritic ecological networks in space: An integrated network perspective. Ecology Letters [Internet]. 2013;16(5):707–19. Available from: http://dx.doi.org/10.1111/ele.12084

4. Dent CL, Grimm NB. Spatial heterogeneity of stream water nutrient concentrations over successional

time. Ecology [Internet]. 1999;80(7):2283–98. Available from: http://www.jstor.org/stable/176910

5. Ver Hoef JM, Peterson EE, Theobald D. Spatial statistical models that use flow and stream distance. Environmental and Ecological Statistics. 2006;13:449–64.

6. Cressie N, Frey J, Harch B, Smith M. Spatial prediction on a river network. Journal of Agricultural, Biological, and Environmental Statistics. 2006;11:127–50.

7. Garreta V, Monestiez P, Ver Hoef JM. Spatial modelling and prediction on river networks: Up model, down model or hybrid? Environmetrics [Internet]. 2010;21(5):439–56. Available from: http://dx.doi.org/10.1002/env.995

8. Le ND, Zidek JV. Statistical analysis of environmental space-time processes. New York, NY: Springer; 2006. (Springer series in statistics).

9. Falk MG, McGree JM, Pettitt AN. Sampling designs on stream networks using the pseudo-bayesian approach. Environmental and Ecological Statistics. 2014;21:751–73.

10. Chaloner K, Verdinelli I. Bayesian experimental design: A review. Statistical Science. 1995;10(3):273–304.

11. Mueller P. Simulation-based optimal design. Bayesian Statistics. 1999;6:459–74.

12. Royle JA. Exchange algorithms for constructing large spatial designs. Journal of Statistical Planning and Inference. 2002;100(2):121–34.

13. Evangelou E, Zhu Z. Optimal predictive design augmentation for spatial generalised linear mixed models. Journal of Statistical Planning and Inference. 2012;142(12):3242–53.

14. Som NA, Monestiez P, Ver Hoef JM, Zimmerman DL, Peterson EE. Spatial sampling on streams: Principles for inference on aquatic networks. Environmetrics. 2014;25(5):306–23.

15. Zhu Z, Stein ML. Spatial sampling design for prediction with estimated parameters. Journal of Agricultural, Biological, and Environmental Statistics. 2006;11(1):24–44.

16. Pronzato L, Muller WG. Design of computer experiments: Space filling and beyond. Statistics and Computing. 2012;22.

17. Morris MD, Mitchell TJ. Exploratory designs for computational experiments. Journal of Statistical

Planning and Inference. 1995;43(3):381–402.

18. Mueller P, Berry DA, Grieve AP, Smith M, Krams M. Simulation-based sequential Bayesian design. Journal of Statistical Planning and Inference. 2007;137:3140–50.
