# Supplementary material for: Correction: SSNdesign—An R package for pseudo-Bayesian optimal and adaptive sampling designs on stream networks
Source: PLoS One. 2026 Jan 27;21(1):e0341916. doi: 10.1371/journal.pone.0341916 (PMC12843544; doi:10.1371/journal.pone.0341916)
Supplement: S1 File — (PDF) [file pone.0341916.s001.pdf]

Responses to an editor’s comments on the corrected Table 1, with annotated code

Alan R. Pearse

1 Preamble

This document is a slightly modified version of a document prepared in response to an editor’s careful review of the corrections to Table 1 of the article, “SSNdesign—An R package for pseudo-Bayesian optimal and adaptive sampling designs on stream networks” (PONE0.238422). The two main purposes of this document are (1) to clarify some questions raised by the editor about the corrections to Table 1, and (2) to provide detailed annotations of the code associated with the original article to show that the SSNdesign R package has always implemented the correct versions of the utility functions as described in the corrected version of Table 1.

The structure of this document is as follows. In Section 2, I establish the notation used throughout my responses to the comments. This includes background on spatial stream network models and mathematical details that are referred to in other parts of the document. In Section 3, I respond to the editor’s comments. I write out the editor’s comments on Table 1 as a series of numbered dot points in bold font. Every comment is followed by a written response. In Section 4, I present annotated code from SSNdesign (https://github.com/apear9/SSNdesign/) to demonstrate that the code at submission of the original manuscript implemented the correct versions of the utility functions, despite the typographical errors in the original version of Table 1, which in turn demonstrates that the results in the paper are not affected by the corrections to Table 1.

2 Notation

This section sets up notation. Section 2.1 explains the referencing convention. Section 2.2 establishes the mathematical symbols needed to discuss spatial stream network models.

2.1 Non-mathematical notation

In Pearse et al. (2020), the editor’s comments, and in this document, “Ref. 15” refers to Som et al. (2014), and “Ref. 16” refers to Falk et al. (2014). Similarly, “Ref. 31” refers to Morris & Mitchell (1995), and “Ref. 32” refers to Pronzato & Mueller (2012). The numbering of each reference

corresponds to the number given to each reference in the original article. The full citations can be found at the end of this document in the reference list.

Equation references relating to other papers (e.g., Ref. 15 and Ref. 16) will be displayed as (for example) "Eq. (10) of Ref. 15" to avoid ambiguity about equations with the same equation number across multiple sources. Equation numbers for equations written in this document are preceded by "R" for "Response". For example, the first numbered equation in this document is labelled Eq. (R1).

2.2 Mathematical notation

Here, we develop the notation to describe spatial stream network (SSN) models. The notation below is consistent with that used throughout Pearse et al. (2020). On a personal note, this article represents my pre-PhD research. Knowing what I know now, I would have written the article differently and used different symbols. But, in this document, I honour the notation of the original article to enable cross-referencing of the equations I write here with the original article, the corrected Table 1, and the editor's comments.

Let $y(\cdot) = \{y(s) : s \in \mathcal{D}\}$ be a spatial stochastic process (e.g., Cressie, 1993, Ch. 2) defined at locations s in spatial domain $\mathcal{D}$. For a stream network, $\mathcal{D}$ is the set of points on one or more dendritic stream networks. The exact topological requirements are described by Peterson and Ver Hoef (2014). For any location $s \in \mathcal{D}$, the spatial process $y(\cdot)$ is modelled as,

$$y(s) = x(s)^{\top}\beta + z(s), \tag{R1}$$

where $x(s)^{\top}$ is an m-variate row vector of covariates at location $s \in \mathcal{D}$, where the "T" denotes a vector/matrix transpose; β is an m-variate column vector of linear-model coefficients (including an intercept) corresponding to the covariates; and $z(\cdot)$ is a mean-zero spatial process equipped with covariance function $\mathcal{C}(s_i, s_j; \theta) \equiv \mathrm{Cov}\left(y(s_i), y(s_j)\right)$, where θ is a vector of covariance parameters.

Following Peterson and Ver Hoef (2010) and Ver Hoef and Peterson (2010), the spatial process $z(\cdot)$, which later becomes the stream-based spatial random effect in a spatial stream network (SSN) model (see below), can be decomposed into 'tail-up' (TU), 'tail-down' (TD), 'Euclidean' (EU), and random-error components of variation. Let $z_{\mathrm{TU}}(\cdot)$ be a mean-zero spatial process with covariance function $\mathcal{C}_{\mathrm{TU}}(s_i, s_j; \theta)$, which represents the TU component of variation in an SSN model. Similarly, and respectively, let $z_{\mathrm{TD}}(\cdot)$, $z_{\mathrm{EU}}(\cdot)$, and $\varepsilon(\cdot)$ be mean-zero spatial processes that represent the TD, EU, and random error components of variation, and let their covariance functions be written as $\mathcal{C}_{\mathrm{TD}}(s_i, s_j; \theta)$, $\mathcal{C}_{\mathrm{EU}}(s_i, s_j; \theta)$, and $\mathcal{C}_{\varepsilon}(s_i, s_j; \theta)$. Refer to Ver Hoef and Peterson (2010) for technical details about the construction of the TU and TD spatial processes and their covariance functions on

stream networks. The EU component is a mean zero, weakly stationary spatial process commonly used in geostatistics (e.g., Cressie, 1993, Ch. 2). The random-error component of variation is a white-noise spatial process that generates independently and identically distributed random variables with no spatial dependence. The components of random variation are assumed to be independent of each other. When all four are present (the most general SSN model), we have, for all $s \in \mathcal{D}$,

$$z(s) = z_{\mathrm{TU}}(s) + z_{\mathrm{TD}}(s) + z_{\mathrm{EU}}(s) + \varepsilon(s).$$

In this case, we can write the covariance function of $y(\cdot)$ as,

$$\mathcal{C}(s_i, s_j; \theta) = \mathcal{C}_{\mathrm{TU}}(s_i, s_j; \theta) + \mathcal{C}_{\mathrm{TD}}(s_i, s_j; \theta) + \mathcal{C}_{\mathrm{EU}}(s_i, s_j; \theta) + \mathcal{C}_{\varepsilon}(s_i, s_j; \theta),$$

for all pairs of locations $s_i, s_j \in \mathcal{D}$. Technical details of these stream-based covariance functions can be found in Peterson and Ver Hoef (2010) and Ver Hoef and Peterson (2010), and some examples are also given in Lines 11-80 of S1 of Pearse et al. (2020).

Let $d = \{s_1, \ldots, s_n\}$ be a design containing n spatial locations. Since the point of optimal design is to plan a future experiment where the data *will be observed*, we do not necessarily observe $y(\cdot)$ at these spatial locations. Nevertheless, we can still consider $\mathrm{Y} = (y(s_1), \ldots, y(s_n))^\top$ as a random vector that has a distribution, mean vector, and covariance matrix, etc. According to the model for the spatial process in (R1), Y follows the spatial linear model,

$$\mathrm{Y} = \mathrm{X}\beta + \mathrm{Z}, \tag{R2}$$

where X is an $n \times m$ matrix of covariates whose i-th row is the row vector $\mathrm{x}(s_i)^\top$, for $i = 1, \ldots, n$, and $\mathrm{Z} = (z(s_1), \ldots, z(s_n))^\top$ is a vector of spatial random effects with mean vector 0 and covariance matrix Σ, whose (i, j) element is calculated as $\mathcal{C}(s_i, s_j; \theta)$ for $i, j = 1, \ldots, n$. Hence, Σ depends on θ, and we can write it as $\Sigma(\theta)$, though the dependence on θ will not be explicitly notated in this document. In the most general SSN model, Z can be decomposed as $\mathrm{Z} = \mathrm{Z}_{\mathrm{TU}} + \mathrm{Z}_{\mathrm{TD}} + \mathrm{Z}_{\mathrm{EU}} + \mathrm{E}$ (i.e., the tail-up, tail-down, Euclidean, and random-error components), where,

- The TU random-effects vector is $\mathrm{Z}_{\mathrm{TU}} = (z_{\mathrm{TU}}(s_1), \ldots, z_{\mathrm{TU}}(s_n))^\top$ with covariance matrix $\mathrm{Var}(\mathrm{Z}_{\mathrm{TU}}) = \Sigma_{\mathrm{TU}}$, whose (i, j) element given by $\mathcal{C}_{\mathrm{TU}}(s_i, s_j; \theta)$ for $i, j = 1, \ldots, n$.
- The TD random-effects vector is $\mathrm{Z}_{\mathrm{TD}} = (z_{\mathrm{TD}}(s_1), \ldots, z_{\mathrm{TD}}(s_n))^\top$ with covariance matrix $\mathrm{Var}(\mathrm{Z}_{\mathrm{TD}}) = \Sigma_{\mathrm{TD}}$ with (i, j) element given by $\mathcal{C}_{\mathrm{TD}}(s_i, s_j; \theta)$ for $i, j = 1, \ldots, n$.
- The EU random-effects vector is $\mathrm{Z}_{\mathrm{EU}} = (z_{\mathrm{EU}}(s_1), \ldots, z_{\mathrm{EU}}(s_n))^\top$ with covariance matrix $\mathrm{Var}(\mathrm{Z}_{\mathrm{EU}}) = \Sigma_{\mathrm{EU}}$ with (i, j) element given by $\mathcal{C}_{\mathrm{EU}}(s_i, s_j; \theta)$ for $i, j = 1, \ldots, n$.

- The random vector $\mathrm{E} = (\varepsilon(s_1), \dots, \varepsilon(s_n))^\top$ is composed of independently and identically distributed errors, each with the same variance, $\sigma_\varepsilon^2 = \mathrm{Var}(\varepsilon(s))$, for all $s \in \mathcal{D}$, so its covariance matrix is $\mathrm{Var}(\mathrm{E}) = \sigma_\varepsilon^2 \mathrm{I}_n$, where I_n is the n-dimensional identity matrix.

As such, we can decompose the covariance matrix $\mathrm{Var}(\mathrm{Y}) = \Sigma$ as

$$\Sigma = \Sigma_{\mathrm{TU}} + \Sigma_{\mathrm{TD}} + \Sigma_{\mathrm{EU}} + \sigma_\varepsilon^2 \mathrm{I}_n.$$

This decomposition is a more general version of the specific example given in Eq. (5) of Ref. 15 (Som et al., 2014), who only consider the TU, TD, and error components.

Suppose we want to predict the spatial process $y(\cdot)$ at a prediction location s_z in the stream network. Best linear unbiased prediction of $y(s_z)$ based on Y is called *kriging* (e.g., Cressie, 1993, Ch. 3). The version of kriging called universal kriging (UK) is appropriate here because, in Eq. (1) and (2), the mean of $y(\cdot)$ is a linear model with unknown coefficients. Universal kriging accounts for the estimation of these unknown coefficients. Let $\mathrm{c}(s_z) = \left(\mathcal{C}(s_z, s_1; \theta), \dots, \mathcal{C}(s_z, s_n; \theta)\right)^\top$ be an n-variate column vector of spatial covariances between the prediction location s_z and the locations $s_1, \dots, s_n$. Then, the universal kriging predictor of $y(s_z)$ based on Y can be written as,

$$\hat{y}(s_z) = \mathrm{x}(s_z)^\top \hat{\beta}_{\mathrm{gls}} + \mathrm{c}(s_z)^\top \Sigma^{-1}\left(\mathrm{Y} - \mathrm{X}\hat{\beta}_{\mathrm{gls}}\right), \qquad \text{(R3)}$$

where $\hat{\beta}_{\mathrm{gls}} = (\mathrm{X}^\top\Sigma^{-1}\mathrm{X})^{-1}\mathrm{X}^\top\Sigma^{-1}\mathrm{Y}$ is the generalised least squares ('gls') estimator of the linear-model coefficients β. Eq. (9) of Ref. 15 (Som et al., 2014) is a rearranged form of this equation. Eq. (4.59) of Cressie & Wikle (2011, p. 148) gives the formula for the universal kriging predictor in the form of (R3).

Since the definitions of $\mathrm{Var}(\hat{\beta}_{\mathrm{gls}})$ and $\mathrm{Var}\left(\hat{\beta}_{\mathrm{gls}}\right)^{-1}$ are a key part of this document and the questions it answers, we give a detailed discussion here. The variance operator $\mathrm{Var}(\cdot)$ gives the variance of a random variable or the covariance matrix of a random vector. For example, we have $\mathrm{Var}(\mathrm{Y}) = \Sigma$ (an $n \times n$ covariance matrix) by construction. An important fact about the variance operator is that, if A is a non-random $a \times n$ matrix (for any positive integer a), then, for example,

$$\mathrm{Var}(\mathrm{AY}) = \mathrm{A}\mathrm{Var}(\mathrm{Y})\mathrm{A}^\top. \qquad \text{(R4)}$$

Since $\mathrm{Var}(\mathrm{Y}) = \Sigma$, we get $\mathrm{Var}(\mathrm{AY}) = \mathrm{A}\Sigma\mathrm{A}^\top$. Hence, by expanding $\hat{\beta}_{\mathrm{gls}}$ inside $\mathrm{Var}(\hat{\beta}_{\mathrm{gls}})$, we see that,

$$\begin{aligned}\mathrm{Var}\left(\hat{\beta}_{\mathrm{gls}}\right) &= \mathrm{Var}((\mathrm{X}^\top\Sigma^{-1}\mathrm{X})^{-1}\mathrm{X}^\top\Sigma^{-1}\mathrm{Y}) \\ &= (\mathrm{X}^\top\Sigma^{-1}\mathrm{X})^{-1}\mathrm{X}^\top\Sigma^{-1}\mathrm{Var}(\mathrm{Y})\Sigma^{-1}\mathrm{X}(\mathrm{X}^\top\Sigma^{-1}\mathrm{X})^{-1},\end{aligned}$$

by applying the rule in Eq. (R4). Substituting $\mathrm{Var}(\mathrm{Y}) = \Sigma$ yields,

$$\mathrm{Var}(\hat{\beta}_{\mathrm{gls}}) = (\mathrm{X}^\top\Sigma^{-1}\mathrm{X})^{-1}\mathrm{X}^\top\Sigma^{-1}\Sigma\Sigma^{-1}\mathrm{X}(\mathrm{X}^\top\Sigma^{-1}\mathrm{X})^{-1}$$
$$= (\mathrm{X}^\top\Sigma^{-1}\mathrm{X})^{-1}(\mathrm{X}^\top\Sigma^{-1}\mathrm{X})(\mathrm{X}^\top\Sigma^{-1}\mathrm{X})^{-1}.$$

After cancelling one $(\mathrm{X}^\top\Sigma^{-1}\mathrm{X})^{-1}$ term with the $(\mathrm{X}^\top\Sigma^{-1}\mathrm{X})$ term on the right-hand side, we obtain,

$$\mathrm{Var}(\hat{\beta}_{\mathrm{gls}}) = (\mathrm{X}^\top\Sigma^{-1}\mathrm{X})^{-1}. \tag{R5}$$

Eq. (R5) is called the covariance matrix of $\hat{\beta}_{\mathrm{gls}}$, and this same expression appears in, for example, Eq. (15) of Ref. 15. A related quantity is the inverse of (R5): That is,

$$\mathrm{Var}(\hat{\beta}_{\mathrm{gls}})^{-1} = \mathrm{X}^\top\Sigma^{-1}\mathrm{X}.$$

The matrix $\mathrm{Var}(\hat{\beta}_{\mathrm{gls}})^{-1}$ is called the Fisher Information Matrix of $\hat{\beta}_{\mathrm{gls}}$.

For some spatial optimal design problems, we may want to compute the universal kriging (UK) variance. Ref. 15 correctly defines the universal kriging variance as $\sigma^2_{\mathrm{UK}}(s_z) = \mathrm{Var}(\hat{y}(s_z) - y(s_z))$ (translated to our notation here). In expanded form, the universal kriging variance at location s_z is written as,

$$\sigma^2_{\mathrm{UK}}(s_z) = \sigma^2_Y - \mathrm{c}(s_z)^\top\Sigma^{-1}\mathrm{c}(s_z) + \left(\mathrm{x}(s_z) - \mathrm{X}^\top\Sigma^{-1}\mathrm{c}(s_z)\right)^\top(\mathrm{X}^\top\Sigma^{-1}\mathrm{X})^{-1}\left(\mathrm{x}(s_z) - \mathrm{X}^\top\Sigma^{-1}\mathrm{c}(s_z)\right), \tag{R6}$$

where $\sigma^2_Y = \mathcal{C}(s_z, s_z; \theta)$ is the marginal variance of the spatial process, and the construction of the stream-based spatial processes in Ver Hoef and Peterson (2010) guarantees this value is constant at all locations on the spatial domain. Note that (R6) appears as Eq. (10) of Ref. 15.

It is possible to calculate the kriging variance simultaneously for all members of a set of prediction locations. Let $S = \{s_{z1}, \dots, s_{zp}\}$ denote a set of p prediction locations. Let V be a $p \times p$ spatial covariance matrix for the prediction locations, $s_{z1}, \dots, s_{zp}$, whose (j,k) element is given by $\mathcal{C}(s_{zj}, s_{zk}; \theta)$ for $j, k = 1, \dots, p$. Let C be a $n \times p$ matrix of spatial covariances between the n locations in the design and the p prediction locations, where the j-th column of the matrix is the column vector $\mathrm{c}(s_{zj}) \equiv \left(\mathcal{C}(s_{zj}, s_1; \theta), \dots, \mathcal{C}(s_{zj}, s_n; \theta)\right)^\top$ for $j = 1, \dots, p$. Also let X_S be an $m \times p$ matrix of covariates at the prediction locations, whose j-th column is the column vector $\mathrm{x}(s_{zj})$ for $j = 1, \dots, p$. Then we can simultaneously calculate the kriging variances at the prediction locations (and the prediction-error covariances between them) by using the formula,

$$\mathrm{V} - \mathrm{C}^\top\Sigma^{-1}\mathrm{C} + (\mathrm{X}_S - \mathrm{X}^\top\Sigma^{-1}\mathrm{C})^\top(\mathrm{X}^\top\Sigma^{-1}\mathrm{X})^{-1}(\mathrm{X}_S - \mathrm{X}^\top\Sigma^{-1}\mathrm{C}). \tag{R7}$$

Every component of Eq. (R7) is a straightforward generalisation of the corresponding term on the right-hand side of Eq. (R6). The diagonal elements of (R7) are $\sigma^2_{\mathrm{UK}}(s_{z1}), \dots, \sigma^2_{\mathrm{UK}}(s_{zp})$, namely the universal kriging variances at prediction locations $s_{z1}, \dots, s_{zp}$.

3 Responses to comments

In this section, the editor's comments are written in numbered dot points with bold font. For clarity, each comment is accompanied by a short annotation in square brackets and normal font that clearly identifies the row of Table 1 to which the editor is referring. My response to each comment is written underneath the relevant dot point. Some responses refer to my previous responses, in which case I refer to the relevant dot point by number. For example, the editor's comment written out in dot point 2. is referred to as **Comment 2** in my other responses.

1. **p. 4, eq 7 ref 16 shows INVERSE (from original) but NO INVERSE is correct. this new version of the equation, with no $^{-1}$, is correct.** [This comment is referring to the CP-optimality row of Table 1.]

Thank you, agreed.

2. **(no changes). ref 15 shows $\mathrm{Var}(\widehat{\boldsymbol{\beta}}_{\mathrm{gls}})$ as the covariance matrix for the estimated beta (beta hat). *the inverse $^{-1}$ does not appear in the equation in ref 15, and the determinant of Var beta hat gls already has the inverse $^{-1}$ in it! SO, i am not sure this was right in the original, and no changes here - it is not possible for me to determine whether it is correct.**

   In this paper's S1, it shows " $\mathrm{Var}(\widehat{\boldsymbol{\beta}}_{\mathrm{gls}})$ ", i.e., without "$^{-1}$", as the covariance matrix, so i am not sure that the authors are including $^{-1}$ correctly HERE, or correctly THERE (in S1). According to ref 15, the $^{-1}$ should NOT be there. [This comment refers to the D-optimality row of Table 1.]

The equation in Table 1 is correct, but the confusion is understandable. In what follows, I show that this equation is entirely consistent with what is written in Ref. 15 (Som et al., 2014) and S1 of Pearse et al. (2020).

The first and most important point to make about Ref. 15 is that, in Ref. 15, optimal designs are found by **minimising loss functions**. On the other hand, Ref. 16 (Falk et al., 2014) and Pearse et al. (2020) construct optimal designs by **maximising utility functions**. Let $L(d, \theta)$ be a loss function, evaluated for design d and parameter values θ. This is a negatively oriented functional of design d that is bounded from below. This simply means smaller values of $L(d, \theta)$ are considered better and

that $L(d, \theta)$ has a minimum. Let $U(d, \theta)$ be a utility function, which is a positively oriented functional of design d that is bounded from above. Again, this simply means that larger values of $U(d, \theta)$ are considered better and that $U(d, \theta)$ has a maximum. Any loss function can easily be turned into a utility function, and *vice versa*. Starting with the loss function $L(d, \theta)$, an equivalent utility function can be constructed by applying a minus sign,

$$U(d, \theta) = -L(d, \theta),$$

or, provided that $L(d, \theta) > 0$ for all designs d and parameter values θ, by taking the inverse,

$$U(d, \theta) = L(d, \theta)^{-1}.$$

As I will show, this resolves most of the issues raised in the comments about minus signs and inverses.

Specifically with respect to D-optimality (as shown in the second row of the corrected Table 1), Ref. 15 defines the **loss function**,

$$L_{\text{D-Som}}(d, \theta) = \det\left(\text{Var}(\hat{\beta}_{\text{gls}})\right), \tag{R8}$$

where $\text{Var}(\hat{\beta}_{\text{gls}}) = (\text{X}^{\top}\Sigma^{-1}\text{X})^{-1}$ per (R5) and "det(·)" is the matrix determinant. The loss function depends on the design d through the matrices X and Σ, and it depends on the covariance parameters θ through Σ. The covariance matrix of $\hat{\beta}_{\text{gls}}$, $\text{Var}(\hat{\beta}_{\text{gls}})$, is *positive definite*, which implies that its inverse, $\text{Var}(\hat{\beta}_{\text{gls}})^{-1} = \text{X}^{\top}\Sigma^{-1}\text{X}$, exists and that $\det(\text{Var}(\hat{\beta}_{\text{gls}})) > 0$.

To convert (R8) into a utility function, we take the inverse of the loss function in (R8), which yields,

$$U_{\text{D-Som}}(d, \theta) = L_{\text{D-Som}}(d, \theta)^{-1} = \det\left(\text{Var}(\hat{\beta}_{\text{gls}})\right)^{-1}.$$

Then, by standard properties of the determinant,

$$\det\left(\text{Var}(\hat{\beta}_{\text{gls}})\right)^{-1} = \det\left(\text{Var}(\hat{\beta}_{\text{gls}})^{-1}\right).$$

This rule is usually recalled by the mnemonic that "the inverse of a determinant is the determinant of the inverse". Therefore,

$$U_{\text{D-Som}}(d, \theta) = \det\left(\text{Var}(\hat{\beta}_{\text{gls}})^{-1}\right).$$

Maximising $U_{\text{D-Som}}(d, \theta)$ is the same as **minimising** $L_{\text{D-Som}}(d, \theta)$ because smaller values of $L_{\text{D-Som}}(d, \theta)$ lead to larger values of $U_{\text{D-som}}(d, \theta)$.

Since $\log(x)$ is a monotone increasing function of $x > 0$, and since $\det\left(\mathrm{Var}(\hat{\beta}_{\mathrm{gls}})^{-1}\right) > 0$, **maximising** $U_{\text{D-Som}}(d,\theta)$ is equivalent to **maximising** the logarithm of $U_{\text{D-Som}}(d,\theta)$. This leads to the D-optimality utility function used in Pearse et al. (2020):

$$U_{\text{D-Pearse}}(d,\theta) = \log\big(U_{\text{D-Som}}(d,\theta)\big) = \log\left(\det\left(\mathrm{Var}(\hat{\beta}_{\mathrm{gls}})^{-1}\right)\right).$$

Therefore, we see that the utility function written in Table 1 is entirely consistent with Ref. 15, even though Ref. 15 does not have the inverse. Therefore, it is correct to have $\mathrm{Var}(\hat{\beta}_{\mathrm{gls}})^{-1}$ in Table 1.

As for S1 of Pearse et al. (2020), please see Eq. (10) of S1 and the sentence below it. The underlined parts of the screenshot from S1 below clearly shows that we do use $\mathrm{Var}(\hat{\beta}_{\mathrm{gls}})^{-1}$ in the definition of the D-optimality utility function.

Our fixed effects estimation utility is called D-optimality (9,14). This utility works on the same principle as CP-optimality, though it minimises the uncertainty in a different set of parameters. Formally, the utility function is

$$U(d,\theta) = \log\det\left[I(d,\beta_{\mathrm{gls}})\right], \qquad (10)$$

where $I(d,\beta_{\mathrm{gls}}) = Var(\beta_{\mathrm{gls}}, d)^{-1} = X^T\Sigma^{-1}X$ is the Fisher information for the fixed effects parameters.

3. **see above for note about including the inverse $^{-1}$, and this is different from what they use in their own S1. no changes from old to new, and "var hat", it is noted in the table notes, is their estimated variance. (that is why it's Var hat and not Var).** [This comment refers to the ED-optimality row of Table 1.]

Please refer to my response to **Comment 2** to resolve the question about the inverse. Also see Eq. (11) of S1, which uses what is called “the observed Fisher Information Matrix” of $\hat{\beta}_{\mathrm{gls}}$. The Fisher Information Matrix is, by definition, the inverse of the covariance matrix of $\hat{\beta}_{\mathrm{gls}}$; see Section 2 of this document.

4. **see above for term 1 (beta hat, might not be correct to include the $^{-1}$), and above for term 2 (no inverse on the 2nd term is correct).** [This comment refers to the CPD-optimality row of Table 1.]

Regarding the issue with 'term 1', please refer to my response to **Comment 2**, which shows that it is correct to include the inverse for this term. We agree about the correctness of the second term.

5. [Both parts of the following comment refer to the K-optimality row of Table 1.]

1. **neither 15 nor 16 uses the "UK" subscript. but, "U" can't be used alone as a subscript because it means "up<stream>" to readers of papers like this, it seems. Other sources (refs 15 and 16 for example) just use the subscript "K" for kriging (without specifying it is universal kriging with a $_{UK}$). Since this paper seems to be talking about "K-optimality", using K alone as a subscript probably would also be confusing. So, the UK subscript seems a logical change**.

On point 1., thank you; that was the intent with the 'UK' subscript. In spatial statistics, there are three commonly used types of kriging, namely simple kriging (SK), ordinary kriging (OK), and universal kriging (UK) (e.g., Cressie, 1993, Ch. 3), so the 'UK' subscript is also intended to provide clarity in this regard.

2. **References 15 (uses "var (y hat(sj))") and 16 (uses sigma squared -without an s reference - and, includes an error term) do NOT give the same equation. Neither the original table, nor this version, include the error term (ref 15: "(var(y)=sigma^2_u + sigma^2_D + *sigma$^2_0$*", i.e., including terms *for* up, down, and error), and ref 16 uses "var (yhat)" for each segment (sj). So it isn't clear why this change was made. the inverse ($^{-1}$) is included in old and new equations, and I can't tell why.**

On point 2., there are several sub-points that I need to address separately.

First, regarding Ref. 15 and "var(y)=sigma^2_u + sigma^2_D + *sigma$^2_0$*", this comment seems to be referring to Eq. (5) of Ref. 15, which gives the decomposition of $\mathrm{Var}(\mathrm{Y})$ (i.e., the covariance matrix of the spatial data) in terms of the tail-up, tail-down, and white-noise error components, which was also discussed in Section 2 of this document. (Eq. (5) of Ref. 16 also happens to describe the same thing, and our version of Eq. (5) of Ref. 15 also appears in our S1 as Eq. (5).) **This is different to the kriging variance, though it is somewhat related because it is involved in the calculation of Σ in Eq. (R6) and V in Eq. (R7).**

The correct equation to look at in Ref. 15 is Eq. (10). On the left-hand side of Eq. (10) in Ref. 15, Som et al. (2014) denote the universal kriging variance by "$\sigma^2_{\mathrm{K}}(s_0)$" for a prediction location s_0, which is consistent with the notation used in the corrected version of Table 1. (The difference is that I prefer

the "UK" subscript, as explained in my response to **Comment 5.1**, and I index the prediction sites as $s_z \in S$.)

Second, regarding Ref. 16 and their use of $\mathrm{Var}(\hat{y}(s_z))$, I apologise for the fact that the change in notation for the universal kriging variance from $\mathrm{Var}(\hat{y}(s_z))$ to $\sigma^2_{\mathrm{UK}}(s_z)$ was not well explained in my corrections form nor obvious from the Table. There is a (very technical) explanation for the change that I was not able to fully outline in the corrections form, because the fields in the form did not allow me to enter equations. I give the full explanation below.

The notation of "$\mathrm{Var}(\hat{y}(s_z))$" that appeared in the original version of Table 1 was adopted from Ref. 16 (Falk et al., 2014, p. 756). However, this choice of notation turns out to be unfortunate. The problem is quite subtle. While Falk et al. (2014) and the original Table 1 of Pearse et al. (2020) are unambiguous in referring to the universal kriging variance (**and, importantly, both implement the correct formulas to calculate it**; see below), the notation of $\mathrm{Var}(\hat{y}(s_z))$ is not, strictly speaking, correct. In mathematical statistics, $\mathrm{Var}(\cdot)$ is an operator that returns the variance of a random variable or the covariance matrix of a random vector. Recalling Eq. (R3), the universal kriging predictor at location s_z is given by,

$$\hat{y}(s_z) = \mathrm{x}(s_z)^\top \hat{\beta}_{gls} + \mathrm{c}(s_z)^\top \Sigma^{-1}\left(\mathrm{Y} - \mathrm{X}\hat{\beta}_{gls}\right).$$

Substituting $\hat{\beta}_{gls} = (\mathrm{X}^\top\Sigma^{-1}\mathrm{X})^{-1}\mathrm{X}^\top\Sigma^{-1}\mathrm{Y}$ and rearranging yields,

$$\hat{y}(s_z) = \left[\mathrm{c}(s_z)^\top\Sigma^{-1} + \left(\mathrm{x}(s_z) - \mathrm{X}^\top\Sigma^{-1}\mathrm{c}(s_z)\right)^\top (\mathrm{X}^\top\Sigma^{-1}\mathrm{X})^{-1}\mathrm{X}^\top\Sigma^{-1}\right]\mathrm{Y}.$$

This is equivalent to Eq. (9) of Ref. 15. Then applying the variance operator yields,

$$\mathrm{Var}\left(\hat{y}(s_z)\right) = \left[\mathrm{c}(s_z)^\top\Sigma^{-1} + \left(\mathrm{x}(s_z) - \mathrm{X}^\top\Sigma^{-1}\mathrm{c}(s_z)\right)^\top (\mathrm{X}^\top\Sigma^{-1}\mathrm{X})^{-1}\mathrm{X}^\top\Sigma^{-1}\right]\mathrm{Var}(\mathrm{Y})\left[\mathrm{c}(s_z)^\top\Sigma^{-1} + \left(\mathrm{x}(s_z) - \mathrm{X}^\top\Sigma^{-1}\mathrm{c}(s_z)\right)^\top (\mathrm{X}^\top\Sigma^{-1}\mathrm{X})^{-1}\mathrm{X}^\top\Sigma^{-1}\right]^\top,$$

according to the standard property of the variance operator shown in Eq. (R4). Making the substitution of $\mathrm{Var}(\mathrm{Y}) = \Sigma$ into the equation above yields,

$$\mathrm{Var}\left(\hat{y}(s_z)\right) = \left[\mathrm{c}(s_z)^\top\Sigma^{-1} + \left(\mathrm{x}(s_z) - \mathrm{X}^\top\Sigma^{-1}\mathrm{c}(s_z)\right)^\top (\mathrm{X}^\top\Sigma^{-1}\mathrm{X})^{-1}\mathrm{X}^\top\Sigma^{-1}\right]\Sigma\left[\mathrm{c}(s_z)^\top\Sigma^{-1} + \left(\mathrm{x}(s_z) - \mathrm{X}^\top\Sigma^{-1}\mathrm{c}(s_z)\right)^\top (\mathrm{X}^\top\Sigma^{-1}\mathrm{X})^{-1}\mathrm{X}^\top\Sigma^{-1}\right]^\top.$$

Expanding out the quadratic form gives,

$$\begin{aligned}\mathrm{Var}\left(\hat{y}(s_z)\right) = {} & \mathrm{c}(s_z)^\top\Sigma^{-1}\mathrm{c}(s_z) + 2\mathrm{c}(s_z)^\top\Sigma^{-1}\mathrm{X}(\mathrm{X}^\top\Sigma^{-1}\mathrm{X})^{-1}(\mathrm{x}(s_z) - \mathrm{X}^\top\Sigma^{-1}\mathrm{c}(s_z)) \\ & + (\mathrm{x}(s_z) - \mathrm{X}^\top\Sigma^{-1}\mathrm{c}(s_z))^\top(\mathrm{X}^\top\Sigma^{-1}\mathrm{X})^{-1}(\mathrm{x}(s_z) - \mathrm{X}^\top\Sigma^{-1}\mathrm{c}(s_z)).\end{aligned}$$

Now compare this to (R6), which gives the equation for the universal kriging variance at location s_z. It is clear that $\mathrm{Var}(\hat{y}(s_z)) \neq \sigma_{UK}^2(s_z)$! The reason for this discrepancy is that $\mathrm{Var}(\hat{y}(s_z))$ denotes ***the variance of the kriging predictor***, which is not the same as the ***kriging variance***.

The **variance of the kriging predictor** and the **kriging variance** are related, however. In Ref. 15, above their Eq. (10), Som et al. (2014) correctly define the universal kriging variance (in their notation) as $\sigma_{\mathrm{K}}^2(s_0) = \mathrm{Var}(\hat{y}(s_0) - y(s_0))$. In our notation, the equivalent definition is $\sigma_{\mathrm{UK}}^2(s_z) = \mathrm{Var}(\hat{y}(s_z) - y(s_z))$. Following standard rules for the variance, this can be expanded as,

$$\mathrm{Var}(\hat{y}(s_z) - y(s_z)) = \mathrm{Var}(\hat{y}(s_z)) + \mathrm{Var}(y(s_z)) - 2\mathrm{Cov}(\hat{y}(s_z), y(s_z)),$$

which shows that $\mathrm{Var}(\hat{y}(s_z))$, the **variance of the kriging predictor**, is only one component of the kriging variance. Therefore, the point of updating the notation to "$\sigma_{\mathrm{UK}}^2(s_z)$" in Table 1 is to avoid the confusion between the **variance of the kriging predictor** and the **kriging variance**, which are different, albeit related, quantities.

Despite the notational problems, I want to stress that there was **nothing wrong with the calculation of the kriging variance** in Falk et al. (2014) and Pearse et al. (2020). Falk et al. (2014) refer to Cressie (1993), who unmistakably gives the correct formula for the universal kriging variance on p. 155 of his book. Therefore, they were almost certainly using the right formula to calculate the kriging variance. For Pearse et al. (2020), the formula for the kriging variance written in Table 1 of S1 is exactly Eq. (R7) of this document. Further, an analysis of the code for K-optimality (see the end of this document) demonstrates that we were using the correct equation to calculate the universal kriging variance. The only problem here is that we made a bad choice of symbols to denote the kriging variance in Table 1.

As for the question about the inverse, the inverse is included because we want a utility function that can be **maximised**. Smaller kriging variances translate to lower prediction uncertainty, so it is desirable to **minimise** the sum (or average) of the kriging variances. That is,

$$L_{\mathrm{K}}(d, \theta) = \sum_{s_z \in S} \sigma_{\mathrm{UK}}^2(s_z),$$

is a loss function that must be **minimised**. Ref. 15 mention this loss function (in words only) below their Eq. (11). Following the principles of converting a loss function into a utility function given in my response to **Comment 2**, we can define a utility function based on the sum of the kriging variances by taking the inverse of the sum. That is, as shown in Table 1,

$$U_{\mathrm{K}}(d,\theta) = \left(\sum_{s_z \in S} \sigma^2_{\mathrm{UK}}(s_z)\right)^{-1},$$

is a utility function that must be **maximised**. This leads directly to our equation in Table 1. This exact utility function is also the first equation on p. 756 of Ref. 16 (Falk et al., 2014), though we have changed the symbol for the universal kriging variance from $\mathrm{Var}(\hat{y}(s_z))$ to $\sigma^2_{\mathrm{UK}}(s_z)$ for the reasons given above.

6. **see note above; also, $\hat{\sigma}^2_{\mathrm{UK}}$ (sigma^2 hat) is the estimated variance; so this table uses that ("$\hat{\sigma}^2_{\mathrm{UK}}$") and also "$\widehat{\mathrm{Var}}$"(Var hat) - so I don't know why they switch. again, i couldn't find this equation in either ref 15 or 16.** [This comment refers to the EK-optimality row of Table 1.]

This table uses both $\hat{\sigma}^2_{\mathrm{UK}}$ (the universal kriging variance based on estimated covariance parameters) and $\widehat{\mathrm{Var}}$ because of the issue with the notation outlined in my response to **Comment 5.2**. That is, it makes sense to refer to $\widehat{\mathrm{Var}}$ when discussing the covariance matrix of the fixed effects parameters since we are directly applying the variance operator to $\hat{\beta}_{\mathrm{gls}}$, but the notation of $\sigma^2_{\mathrm{UK}}(s_z)$ and $\hat{\sigma}^2_{\mathrm{UK}}(s_z)$ is more appropriate for the kriging variances since the application of the variance operator in this case is less straightforward.

As for where this function appears in the cited references, Ref. 16 (Falk et al., 2014) define this exact utility function in the paragraph below the first displayed equation of p. 756, when they refer to "$u_{Epred}(d,\theta)$" (their notation). Ref. 15 (Som et al., 2014) refer to a similar empirical-prediction loss function in Eq. (12) and (13) of their paper, where they define the "EK-optimality" loss function.

7. **In this paper's S1, it shows "$\mathrm{Var}(\hat{\beta}_{\mathrm{gls}})$ ", i.e., without "$^{-1}$"in the exponent, the covariance matrix, so i am not sure that the authors are including $^{-1}$ correctly HERE, or correctly THERE (in S1). According to ref 15, the $^{-1}$ should NOT be there.**

   I think it is correct to have omitted the $^{-1}$ on the theta term. [This comment refers to the sequential D-optimality row of Table 1.]

For the first part of the comment, please refer to my response to **Comment 2**. Also, in Lines 247-253 of S1, we are clear that the Sequential D-optimality utility uses the Fisher Information Matrix, which is, by definition, equivalent to $\mathrm{Var}(\hat{\beta}_{\mathrm{gls}})^{-1}$. Therefore, $\mathrm{Var}(\hat{\beta}_{\mathrm{gls}})^{-1}$ is correct. For the second part,

yes, thank you; I agree.

8. **In this paper's S1, it shows "$\mathrm{Var}(\hat{\boldsymbol{\beta}}_{\mathrm{gls}})$ " -i.e., without "$^{-1}$" -as the covariance matrix, so i am not sure that the authors are including $^{-1}$ correctly HERE, or correctly THERE (in S1). According to ref 15, the $^{-1}$ should NOT be there. I think it is correct to have ommitted the $^{-1}$ on the theta term.**

   also - using "var" instead of "sigma^2" suggests you're estimating the variance (sigma^2 is the true population value) with "var". So i don't know what is the difference between "var" and "var hat" to the authors. I can't tell why they use both Var hat (estimate of "Variance") and sigma^2 hat (literally estimated variance parameter, sigma^2) in this table. [This comment refers to the sequential ED-optimality row of Table 1.]

For the first part of the comment, please refer to my response to **Comment 2**, which establishes that the inverse for the first term is correct. Similarly, Lines 247-253 of S1 discuss this utility function and, there, we are clear that the Sequential ED-optimality utility uses the Fisher Information Matrix, which is, by definition, equal to $\widehat{\mathrm{Var}}(\hat{\beta}_{\mathrm{gls}})^{-1}$.

For the second part of the comment, $\mathrm{Var}(\cdot)$ is being used in the sense of the variance operator in mathematical statistics. As explained in the last sentence of the table note attached to the corrected version of Table 1, $\widehat{\mathrm{Var}}(\cdot)$ is not necessarily an "estimated variance", but it is the variance that one calculates after plugging in the estimated values of the covariance parameters $\hat{\theta}$ into the covariance function $C(s_i, s_j; \hat{\theta})$; see Section 2 for a discussion of the covariance function $C(s_i, s_j; \theta)$. Similarly, $\hat{\sigma}^2_{\mathrm{UK}}$ denotes the kriging variance that uses the estimated covariance parameters $\hat{\theta}$.

The use of "σ^2_{UK}" (or at least "σ^2_{K}") to denote the universal kriging variance is the norm in spatial statistics (e.g., Cressie, 1993, p. 155). This should have been used in the first instance but, at the first submission of the paper, I had simply adopted the same symbols as those I saw in Falk et al. (2014), not realising that writing "$\mathrm{Var}(\hat{y}(s_z))$" was problematic for the reasons discussed in my response to **Comment 5.2**.

9. **changing x to s makes sense since s is the indicator for space or segment (used across references) -but S (capital S) is noted in this table to represent sites. However, I did not find this expression anywhere in ref 32 (and i went back and forth through it 5 times). Importantly, i don't know which one of these (S, s, or x) their R package is using. If the**

package includes x, but the equation requires s (which is also maybe specified in a data set), then the equations will be very different unless all the x values are equivalent to the s values! X is the design matrix (according to S1) - so changing the R package from x to s would have significant effects. [This comment refers to the Maximin row of Table 1.]

The equation in Table 1 comes from the second displayed equation on the second page (p. 682 in journal pagination) of Ref. 32 (Pronzanto & Mueller, 2012). However, they write "$\phi_{Mm}(\xi) = \min_{i \neq j} d_{ij}$", and they denote the distance between point i and point j as d_{ij}, whereas I explicitly write $D(s_i, s_j)$ for the distance between points s_i and s_j in the design. The sentence above this equation in Ref. 32 explicitly confirms that this expression is a utility function (i.e., they write that this criterion is "**to be maximized**", emphasis mine), so it can be directly translated into our maximisation-based framework without modification.

As for the question of x and s, I apologise for the confusion. The maximin utility function in Table 1 has always referred to the distances between spatial locations, and this is confirmed by the original table note for Table 1. However, using x_i and x_j to denote spatial locations in the original version of Table 1 turned out to be a very poor choice on my part. In the original Table 1, I made this choice because Ref. 32 used the symbols x_i and x_j to denote spatial locations. This was fine for them, but I should have been more careful when translating the utility function to our paper. As you have rightly pointed out, 'x' suggests a covariate and 's' suggests a spatial location. When preparing the corrections for the other utility functions, I recognised this problem, and I wanted to take the opportunity to update the notation. Now the corrected version of Table 1 uses s_i and s_j to denote the spatial locations instead, which is less ambiguous and more consistent with the rest of the paper.

Below, I present annotated code from the R package to show that the code has always (correctly) used the s values (i.e., the spatial locations), or rather the distances between the spatial locations. The code (https://github.com/apear9/SSNdesign/blob/master/R/spaceFillingMaxiMin.R) is as follows. The function uses a pre-computed distance matrix (using the spatial locations in the design) to evaluate the utility function.

```
spaceFillingMaxiMin <- function(ssn, glmssn, design.points, prior.parameters, n.draws, extra.arguments){

  d <- extra.arguments$Matrices.Obs$d
  ij <- row.names(d) %in% design.points
  d <- d[ij, ij]
  v <- d[upper.tri(d)]

  return(min(v[v != 0])[1])

}
```

Extracts a distance matrix (based on spatial locations)

Subsets the distance matrix to include only entries associated with the design under consideration

Extracts all upper-triangular elements into a vector (v). Note that the upper-triangular elements are the same as the lower-triangular elements. Extracting the upper-triangular elements also excludes the diagonal elements of the distance matrix, which are all zero.

This last line returns the minimum distance between non-collocated sites in the design.

10. **not only has the ^{p} changed to $^{-p}$ comparing the old to the new version, making this raised to p power instead of take the pth root, but also, the new equation applies this (take the pth root) to JUST the D terms. This is a general match to equation 2.1 of ref 31, appearing on p. 384 of that manuscript. However, in this table. "D_W" refers to " the w-th smallest of the W unique non-zero distances between pairs of sites in a design ", while in ref 31, "D" refers to " a maximin design" (p. 383). in Ref 31, this equation appears as a function (phi$_p$) of D, ϕ_p(D), and J is an index list (in ref 31) vs. "the number of unique pairs of sites separated by the distance D_w". In this table, the expression has a minus sign; in equation 2.1 of ref 31, there is no minus sign.** Just **in case this expression is meant to represent equation 2.1 of ref 31, this edit is correct - the exponent on ONLY Dw *should* both a) only apply to the D term; and b) should be $^{-p}$ and not ^{p}. I don't know why there's a minus sign; that doesn't appear in ref 31 anywhere.** [This comment refers to the Morris-Mitchell row of Table 1.]

Yes, this equation is based on Eq. (2.1) of Ref. 31 (Morris & Mitchell, 1995). Everything but the minus sign matches Eq. (2.1). As for the minus sign, Eq. (2.1) defines a loss function that needs to be **minimised**, and Morris & Mitchell (1995) confirm this by writing that "the designs that **minimize** ϕ are the [Maximin] designs in that class" (emphasis mine). Therefore, we applied the minus sign to turn this loss function into a utility function, in line with the principles explained in my response to **Comment 2**.

For peace of mind, I have also annotated the code for the Morris-Mitchell utility function in SSNdesign (https://github.com/apear9/SSNdesign/blob/master/R/spaceFillingMorrisMitchell.R). See below that the original implementation of this utility function was correct.

```
spaceFillingMorrisMitchell <- function(ssn, glmssn, design.points, prior.parameters, n.draws, extra.arguments){

  d <- extra.arguments$Matrices.Obs$d          Distance matrix
  p <- extra.arguments$p                       User-specified power parameter
  ij <- row.names(d) %in% design.points        This code subsets the design matrix to only those entries associated
  d <- d[ij, ij]                               with the design under evaluation.
  v <- d[upper.tri(d) & d != 0]
  ds <- sort(unique(v))          This is D_w
  dsp <- ds^-p                  This is D_w^(-p)
  J <- numeric(length(ds))
  for(i in 1:length(ds)){
    J[i] <- sum(ds == ds[i])                   This finds the number of times the distance occurs in the upper triangle of
  }                                            the distance matrix.

  return(-sum((J * dsp))^(1/p))                This equation matches the equation in the corrected Table 1.

}
```

4 Highlighted code to show that results are not affected

In what follows, I demonstrate that the results in Pearse et al. (2020) are not affected by the typographical errors in Table 1 by showing that the functions from the SSNdesign R package that were used to compute the results in the paper have always implemented the correct versions of the utility functions.

In the Lake Eacham case study, the CPD-optimality and K-optimality utility functions are used. In the Pine River case study, only K-optimality is used. Therefore, the results are correct and unaffected by the corrections to Table 1 if CPD-optimality and K-optimality were correctly implemented at submission. I present highlighted code to demonstrate that this is the case.

Per Row 4 of the corrected Table 1, CPD-optimality is the sum of CP-optimality and D-optimality. Consequently, the R function for CPD-optimality in SSNdesign calls the functions for CP-optimality and D-optimality; see below. Therefore, the CPD-optimality function is correct if CP-optimality and D-optimality are correctly implemented.

```
#' @inherit DOptimality
#' @export
CPDOptimality <- function(ssn, glmssn, design.points, prior.parameters, n.draws, extra.arguments){

  CPOptimality(ssn, glmssn, design.points, prior.parameters, n.draws, extra.arguments) + DOptimality(ssn, glmssn, design.points, prior.parameters, n.draws, extra.arguments)
}
```

CP-optimality (first row in Table 1) **plus** **D-optimality (second row in Table 1)**

The correctness of CP-optimality is verified below. The code has been taken from the SSNdesign GitHub repository (https://github.com/apear9/SSNdesign/blob/master/R/CPOptimality.R), and this function has not been updated since I submitted the manuscript in late 2019.

```
CPOptimality <- function(ssn, glmssn, design.points, prior.parameters, n.draws, extra.arguments){

  # Get design matrix
  ind <- row.names(extra.arguments$obs.X) %in% design.points
  X <- extra.arguments$obs.X[ind, ]
  Xt <- t(X)

  # Get coord information
  ind <- row.names(extra.arguments$obs.C) %in% design.points
  cds <- extra.arguments$obs.C[ind, ]

  ## Get distance matrices, etc.
  mat <- extra.arguments$Matrices.Obs
  ind.mat <- row.names(mat$d) %in% design.points
  mat$d <-  mat$d[ind.mat, ind.mat]
  mat$a <-  mat$a[ind.mat, ind.mat]
  mat$b <-  mat$b[ind.mat, ind.mat]
  mat$w <-  mat$w[ind.mat, ind.mat]
  n.zero <- extra.arguments$net.zero.obs[ind.mat, ind.mat]
```

This code extracts the matrix of covariates at the locations in the design.

This code extracts the coordinates of the locations in the design.

This code extracts and subsets the distance matrices associated with the points in the design.

```r

    ## Simulate covariance parameters
    # cvp.cols <- length(glmssn$estimates$theta)
    # cvp <- matrix(nrow = n.draws, ncol = cvp.cols)

    ## Get other model parameters
    td <- glmssn$args$useTailDownWeight
    cm <- glmssn$args$CorModels
    un <- glmssn$args$use.nugget
    ua <- glmssn$args$use.anisotropy
    re <- glmssn$sampInfo$REs

```

This code is commented out and does nothing.

This code extracts options and settings from an argument of the function.

```r
    ## Perform MC simulations
    FIM <- vector("numeric", n.draws)

    # Before we enter the loop, make sure the step size for ffd is defined
    if(is.null(extra.arguments$h)){
      h <- 1e-5
    } else {
      h <- extra.arguments$h
    }

```

This code initialises a vector to store results.

This is the step size for finite forward differences, which is used to numerically compute the derivatives of a matrix. (This is needed later to calculate the expected Fisher Information Matrix of the covariance parameters.)

```r
    for(i in 1:n.draws){

      # Get covariance matrix on the data
      theta.i <- prior.parameters[i, ]
      V <- SSN:::makeCovMat(
        theta.i,
        mat$d,
        mat$a,
        mat$b,
        mat$w,
        n.zero,
        cds[, "x"],
        cds[, "y"],
        cds[, "x"],
        cds[, "y"],
        td,
        cm,
        un,
        ua,
        re
      )
      Vi <- solve(V)

```

This begins a loop.

This code extracts a set of values for the covariance parameters, simulated from prior distributions.

This code uses a helper function from the SSN R package to compute the spatial covariance matrix "Sigma", though, here, it is labelled as "V".

This code computes "Sigma^(-1)".

```
67
68        #       precompute the matrix P
69        P <- Vi - Vi %*% X %*% solve(Xt %*% Vi %*% X) %*% Xt %*% Vi
70
71        # estimate partial derivatives
```

(a) (b) (c) (d)

This code computes the matrix "P" from the equation given by Falk et al. (2014) on p. 756.

$$P = \Sigma^{-1} - \Sigma^{-1}X(X^T\Sigma^{-1}X)^{-1}X^T\Sigma^{-1}$$

(a) (b) (c) (d)

```
71        # estimate partial derivatives
72        np <- length(theta.i) # np = number of parameters
73        ep <- vector("list", np) # empty vector of partial derivatives (hence e p)
74        em <- matrix(0, nrow = np, ncol = np) # empty vector of matrices (hence e m)
75        for(j in 1:np){
76          theta.j <- theta.i
77          theta.j[j] <- theta.i[j] + h
78          V.j <- SSN:::makeCovMat(
79            theta.j,
80            mat$d,
81            mat$a,
82            mat$b,
83            mat$w,
84            n.zero,
85            cds[, "x"],
86            cds[, "y"],
87            cds[, "x"],
88            cds[, "y"],
89            td,
90            cm,
91            un,
92            ua,
93            re
94          )
95          ep[[j]] <- (V.j - V)/h
96        }
```

This code (from Line 75 up to Line 96) numerically calculates the partial derivatives in the equation for the (i,j) element of the Fisher Information Matrix for the covariance parameters, as written on p. 756 of Falk et al. (2014).

I have pasted in a screenshot of the equation below, and I clearly mark what part of the equation is being calculated here.

$$\frac{1}{2}\text{tr}\left(P\frac{\partial\Sigma}{\partial\theta_i}P\frac{\partial\Sigma}{\partial\theta_j}\right)$$

This uses the forward finite difference method to approximate the derivative, numerically.

```
97
98        for(j in 1:np){
99          for(k in j:np){
100           I_REML <- P %*% ep[[j]] %*% P %*% ep[[k]]
101           em[k, j] <- em[j, k] <- 1/2 * sum(diag(I_REML))
102         }
103       }
104       FIM[i] <- log(det(em))
105
106     }
107
108     if(any(is.infinite(FIM)) | any(is.nan(FIM))){
109       return(-1e9)
110     } else{
111       return(mean(FIM, na.rm = TRUE))
112     }
113
114   }
```

(a) (b)

This code computes the Fisher Information Matrix of the covariance parameters under Restricted Error Maximum Likelihood. See below for a comparison of the code to screenshots of the equation given on p. 756 of Falk et al. (2014).

(a) (b)

$$\frac{1}{2}\text{tr}\left(P\frac{\partial\Sigma}{\partial\theta_i}P\frac{\partial\Sigma}{\partial\theta_j}\right)$$

N.B. "tr" means "trace" (the sum of the diagonal elements of a matrix).

Line 104 shows I take log(det(I(theta))) as shown in the corrected version of Table 1. This is the logarithm of the determinant of the Fisher Information Matrix, which is correct.

This code averages over the prior draws to calculate the expected utility.

Below, the D-optimality code (https://github.com/apear9/SSNdesign/blob/master/R/Doptimality.R) is shown to be correct. The code for the utility function has been split into two annotated screenshots due to length. The first of the screenshots is not particularly interesting, and it simply shows a matrix of covariates and a distance matrix being defined for use in later calculations.

```r
DOptimality <- function(ssn, glmssn, design.points, prior.parameters, n.draws, extra.arguments){

  ## Get data for design points. Note that design matrix is stored as obs.X in extra.arguments
  ind <- row.names(extra.arguments$obs.X) %in% design.points
  X <- extra.arguments$obs.X[ind, ]
  Xt <- t(X)

  ## Get coordinates of points in case the covariance function includes a Euclidean dist-based component
  #  Note that the coordinates (with the same ordering as the design matrix) is stored as obs.C in extra.arguments
  ind.cds <- row.names(extra.arguments$obs.C) %in% design.points
  cds <- extra.arguments$obs.C[ind.cds, ] # names are already x and y

  ## Get distance matrices, etc.

  mat <- extra.arguments$Matrices.Obs
  ind.mat <- row.names(mat$d) %in% design.points
  mat$d <-  mat$d[ind.mat, ind.mat]
  mat$a <-  mat$a[ind.mat, ind.mat]
  mat$b <-  mat$b[ind.mat, ind.mat]
  mat$w <-  mat$w[ind.mat, ind.mat]
  n.zero <- extra.arguments$net.zero.obs[ind.mat, ind.mat]

  ## Get other model parameters

  td <- glmssn$args$useTailDownWeight
  cm <- glmssn$args$CorModels
  un <- glmssn$args$use.nugget
  ua <- glmssn$args$use.anisotropy
  re <- glmssn$sampInfo$REs

```

This code extracts and subsets the matrix of covariates associated with the design.

This code extracts the coordinates of the spatial locations in the design.

The second screenshot follows from the first one, and it shows the actual calculation of the utility function. Clearly, the R function calculates $\log(\det(X^\top \Sigma^{-1} X))$, which is equal to $\log\left(\det\left(\mathrm{Var}(\hat{\beta}_{\mathrm{gls}})^{-1}\right)\right)$, as shown in Section 2 and my response to **Comment 2**. Hence the implementation of D-optimality was correct at submission.

```
102     ## Perform MC simulations
103
104     D <- vector("numeric", n.draws)
105
106     for(i in 1:n.draws){
107
108       theta.i <- prior.parameters[i, ]
109       V <- SSN:::makeCovMat(
110         theta.i,
111         mat$d,
112         mat$a,
113         mat$b,
114         mat$w,
115         n.zero,
116         cds[, "x"],
117         cds[, "y"],
118         cds[, "x"],
119         cds[, "y"],
120         td,
121         cm,
122         un,
123         ua,
124         re
125       )
126       covbi <- Xt %*% solve(V) %*% X
127
128       D[i] <- log(det(covbi))
129       # Note that this is the same as -log(det(solve(covbi))) since the inverse of a determinant is the determinant of the inverse
130       # Losing the inversion saves time, especially for large numbers of Monte Carlo draws.
131
132     }
133
134     if(any(is.infinite(D))){
135       return(-1e9)
136     } else {
137       return(mean(D, na.rm = TRUE))
138     }
139
140   }
```

This sets up a numeric vector to store the results.

For each of n.draws iterations,

This codeblock (Lines 108-125) constructs the spatial stream network covariance matrix using the values of the covariance parameters simulated from their prior distributions.

Line 126 computes the Fisher information matrix of the fixed effects. That is, "var(beta_hat)^-1".

Line 128 calculates its log-determinant (i.e., log(det(var(beta_hat)^-1))).

The expected utility is the average value across the n.draws iterations.

Since CP-optimality and D-optimality are both correct (and were correct at submission), the implementation of CPD-optimality used to obtain the results in Pearse et al. (2020) is correct (and was correct at submission). Therefore, the results in this part of the paper are not affected by the typographical errors in Table 1.

Finally, checking K-optimality is straightforward. My changes to Table 1 with respect to K-optimality only affect the notation (i.e., the symbols used to represent the kriging variance). The calculations were correct at submission. I annotate the code below to confirm this. Only the last screenshot contains the calculations. The other code snippets compute intermediate outputs.

```r
3   KOptimality <- function(ssn, glmssn, design.points, prior.parameters, n.draws, extra.arguments){
4
5     ## Get info for the obs sites
6     ind <- row.names(extra.arguments$obs.X) %in% design.points
7     X <- extra.arguments$obs.X[ind, ]
8     Xt <- t(X)
9
10    ## Get coordinates
11    ind.cds <- row.names(extra.arguments$obs.C) %in% design.points
12    cds.obs <- extra.arguments$obs.C[ind.cds, ]
13
14    ## Cut down matrices involving the observations
15
16    mat <- extra.arguments$Matrices.Obs
17    ind.mat <- row.names(mat$d) %in% design.points
18    mat$d <-  mat$d[ind.mat, ind.mat]
19    mat$a <-  mat$a[ind.mat, ind.mat]
20    mat$b <-  mat$b[ind.mat, ind.mat]
21    mat$w <-  mat$w[ind.mat, ind.mat]
22    extra.arguments$Matrices.Obs <- mat
23    net.zero.obs <- extra.arguments$net.zero.obs[ind.mat, ind.mat]
```

This creates an indicator vector used to subset other matrices.

Subset the matrix of covariates to only contain locations in the design.

This extracts and subsets the coordinates of the locations in the design. (These are potentially needed later to calculate spatial covariances.)

This code extracts and subsets several distance matrices associated with the locations in the design.

```r
24
25    # Do the same for the pxo matrix
26    mat <- extra.arguments$Matrices.pxo
27    mat$d <-  mat$d[ind.mat, ]
28    mat$a <-  mat$a[ind.mat, ]
29    mat$b <-  mat$b[ind.mat, ]
30    mat$w <-  mat$w[ind.mat, ]
31    extra.arguments$Matrices.pxo <- mat
32    net.zero.pxo <- extra.arguments$net.zero.pxo[ind.mat, ]
33    # Get info for the pred sites
34    indp <- row.names(extra.arguments$prd.X) %in% ssn@predpoints@SSNPoints[[1]]@point.data$pid
35    X0 <- t(extra.arguments$prd.X[indp, ]) # have this extracted and put in extra.arguments
36    cds.prd <- extra.arguments$prd.C[indp, ]
```

This code extracts and subsets several distance matrices containing distances between the locations in the design and all the prediction locations used to evaluate the K-optimality utility function.

This code extracts the covariate matrix and coordinates of the prediction locations.

Line 35 gets the matrix "X_s" in Eq. (R7).

```r
37
38    # Matrices
39
40    ## Get other model parameters
41    td <- glmssn$args$useTailDownWeight
42    cm <- glmssn$args$CorModels
43    un <- glmssn$args$use.nugget
44    ua <- glmssn$args$use.anisotropy
45    re <- glmssn$sampInfo$REs
46
47    ## Loop here to find utilities across all simulations
48
49    # initialise empty vector to store results
50
51    K_all <- vector("numeric", n.draws)
52
```

This code extracts information relating to the stream-based covariance structures that are relevant for the utility function.

This code initialises a vector to store the results.

```
for(i in 1:n.draws){

  # get simulated covariance parameters

  theta.i <- prior.parameters[i,]

  # get W and Wi

  W <- SSN:::makeCovMat(
    theta.i,
    extra.arguments$Matrices.Obs$d,
    extra.arguments$Matrices.Obs$a,
    extra.arguments$Matrices.Obs$b,
    extra.arguments$Matrices.Obs$w,
    net.zero.obs,
    cds.obs[, "x"],
    cds.obs[, "y"],
    cds.obs[, "x"],
    cds.obs[, "y"],
    td,
    cm,
    un,
    ua,
    re
  )
  Wi <- solve(W)
```

This code extracts a single set of values for the covariance parameters, simulated from the prior distributions on the parameters.

This code forms the spatial covariance matrix for the locations in the design.

This is equivalent to the matrix "Sigma" in our notation.

I cannot recall why I used "W" in the code instead of "Sigma".

I assume I wrote "W" here instead of "Sigma" because
1. it is different to "V" (used later)
2. the name was shorter and easier to write out in subsequent calculations.

This gets "Sigma^(-1)".

```
  V <- SSN:::makeCovMat(
    theta.i,
    extra.arguments$Matrices.prd$d,
    extra.arguments$Matrices.prd$a,
    extra.arguments$Matrices.prd$b,
    extra.arguments$Matrices.prd$w,
    extra.arguments$net.zero.prd,
    cds.prd[, "x"],
    cds.prd[, "y"],
    cds.prd[, "x"],
    cds.prd[, "y"],
    td,
    cm,
    un,
    ua,
    re
  )
```

This code computes the spatial covariance matrix for the prediction locations.

This is the matrix "V" in Eq. (R7) of this document or Table 1 of S1.

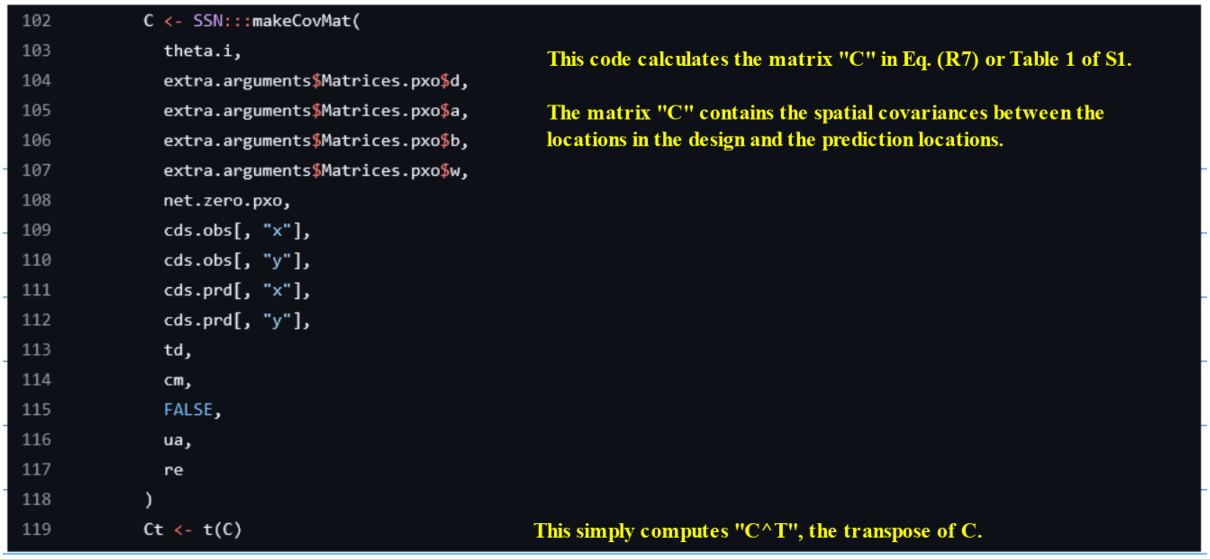

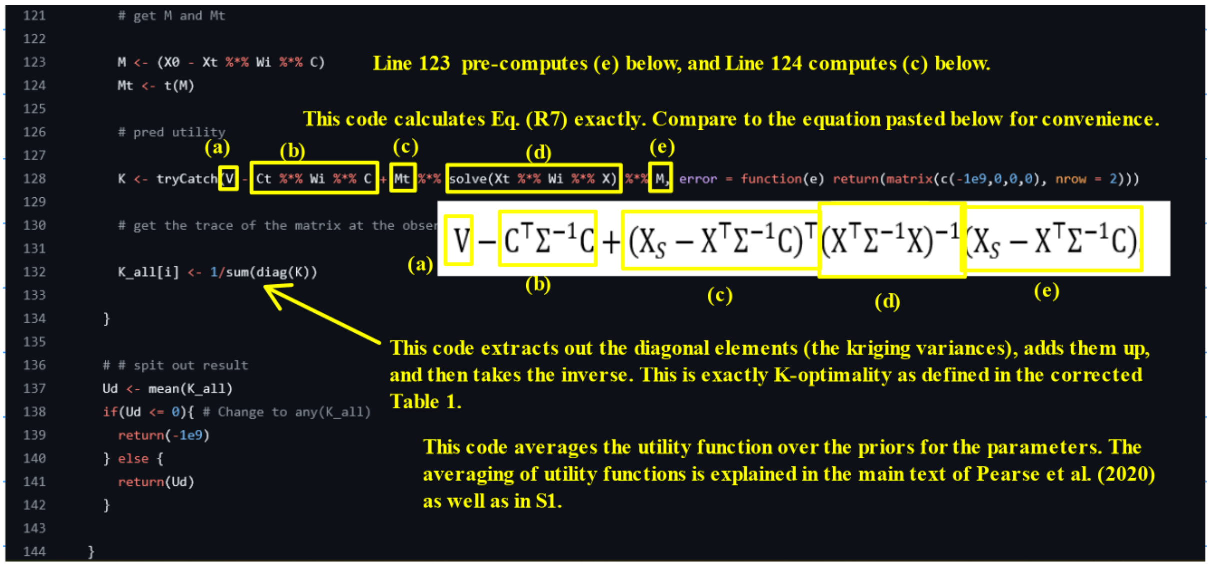

This confirms K-optimality was correct at submission. Therefore, the results in the paper that rely on K-optimality in the paper are correct, and they are unaffected by the notational changes to K-optimality in Table 1.

All of these analyses together demonstrate that CPD-optimality and K-optimality were correctly implemented at submission. Since these were the only utility functions to be used in the case studies presented in the paper, it follows that the empirical results of the paper are sound and are not affected by the changes to Table 1.

References

Cressie, N. (1993). *Statistics for Spatial Data* (rev. ed). Wiley: New York, NY.

Cressie, N., and Wikle, C.K. (2011). *Statistics for Spatio-Temporal Data*. Wiley: Hoboken, NJ.

Falk, M.G., Pettitt, A.N., and McGree, J.M. (2014). Sampling designs on stream networks using the pseudo-Bayesian approach. *Environmental and Ecological Statistics*, *21*, 751-773.

Morris, M.D., and Mitchell, T.J. (1995). Exploratory designs for computational experiments. *Journal of Statistical Planning and Inference*, *142*(12), 3242-3252.

Pearse, A.R., McGree, J.M., Som, N.A., Leigh, C., Maxwell, P., Ver Hoef, J.M., and Peterson, E.E. (2020). SSNdesign – An R package for pseudo-Bayesian optimal and adaptive sampling designs on stream networks. *PLOS One*, *15*(9), e0238422.

Peterson, E.E., and Ver Hoef, J. (2010). A mixed-model moving-average approach to geostatistical modelling in stream networks. *Ecology*, *91*(3), 644-651.

Peterson, E.E., and Ver Hoef, J.M. (2014). STARS: An ArcGIS toolset used to calculate the spatial information needed to fit spatial statistical models to stream network data. *Journal of Statistical Software*, *56*(2), 1-17.

Pronzanto, L., and Mueller, W.G. (2012). Design of computer experiments: space filling and beyond. *Statistics and Computing*, *22*, 681-701.

Som, N.A., Monestiez, P., Ver Hoef, J.M., Zimmerman, D.L., and Peterson, E.E. (2014). Spatial sampling on streams: principles for inference on aquatic networks. *Environmetrics*, 25, 306-323.

Ver Hoef, J., and Peterson, E.E. (2010). A moving average approach for spatial statistical models of stream networks. *Journal of the American Statistical Association*, *105*, 6-18.
